# Correction: Comprehensive techno-environmental evaluation of an isolated PV/wind/biomass hybrid microgrid employing various battery technologies: A comparative analysis

**DOI:** 10.1371/journal.pone.0322420

**Published:** 2025-04-08

**Authors:** The PLOS One Staff

## Notice of Republication

This article was republished on March 21, 2025, to correct errors in the title and funding statement that were introduced during the typesetting process. The publisher apologizes for these errors. Please download this article again to view the correct version. The originally published, uncorrected article and the republished, corrected articles are provided here for reference.

## Supporting information

S1 File Originally published, uncorrected article. (PDF)

S2 File Republished, corrected article. (PDF)
