# Supplementary material for: Comprehensive techno-environmental evaluation of an isolated PV/wind/biomass hybrid microgrid employing various battery technologies: A comparative analysis
Source: PLoS One. 2025 Feb 20;20(2):e0317757. doi: 10.1371/journal.pone.0317757 (PMC11841891; doi:10.1371/journal.pone.0317757)
Supplement: S1 File — (DOCX) [file pone.0317757.s001.docx]

**Table 1:** Estimated Bio-energy Potential from Various Biomass Sources in the Kingdom of Saudi Arabia for the Years 2014 and 2034

| **Biomass source** | **Million ton (estimated)** | **Bio-energy in 2014, mtoe** | **Bio-energy in 2034, mtoe** |
| --- | --- | --- | --- |
| Municipal solid wastes | 10.5 | 3.6 | 4.7 |
| Agricultural crops and residues | 3.5 | 1.2 | 1.7 |
| Forestry crops and residues | 0.5 | 0.2 | 0.3 |
| Agro-industrial residues | 0.8 | 0.3 | 0.5 |
| Sewage (water-carried waste) | 2.5 | 0.5 | 0.8 |
| **Total biomass** | 17.8 | 5.8 | 8 |

**Table 1:** [**https://doi.org/10.1080/15567249.2014.996303**](https://doi.org/10.1080/15567249.2014.996303)

**Table 2.** Technical and economic characteristics of the system components

|  | **Value** | **Unit** |
| --- | --- | --- |
| **PV System** | | |
| cost | 14854 | $/$m^{2}$ |
| Temperature coefficient | 0.0037 | - |
| Efficiency | 15 | % |
| NOCT | 25 | ºC |
| Standard radiation | 1000 | ${W/m}^{2}$ |
| lifetime | 20 | year |
| Replacement cost | 13885 | $ |
| **WT System** | | |
| Rated power | 30 | kw |
| Hub height | 50 | m |
| Efficiency | 80 | % |
| Cut in speed | 2.5 | m/s |
| Rated speed | 12 | m/s |
| Cut-off speed | 25 | m/s |
| lifetime | 20 | year |
| Cost | 3200 | $/kw |
| **Inverter** | | |
| $\mathrm{Efficiency}$ | 95 | % |
| lifetime | 10 | year |
| cost | 711 | $/kw |
| Replacement cost | 650 | $/kw |
| **Biomass Generator** | | |
| $\mathrm{LHV}_{B}$ | 14.8 | MJ/kg |
| $\mathrm{LHV}_{\mathrm{pg}}$ | 4.766 | MJ/kg |
| $B_{\mathrm{rated}}$ | 72 | kg/h |
| $\eta_{g}$ | 80 | % |
| $\mathrm{PG}_{\mathrm{rated}}$ | 40 | KW |
| ($F_{0}$) | 0.0644 | kg/h/50kW |
| $(F_{m})$ | 0.2998 | kg/h/50kW |
| ($\mathrm{BG}_{\mathrm{cost}}$) | 16000 | $/kw |
| lifetime | 20 to 30 | years |

**Table 2.**

[**https://doi.org/10.1109/ACCESS.2019.2936656**](https://doi.org/10.1109/ACCESS.2019.2936656)

[**https://doi.org/10.3390/app112110191**](https://doi.org/10.3390/app112110191)

[**https://doi.org/10.3390/en14020489**](https://doi.org/10.3390/en14020489)
