# Supplementary material for: Correction: Comprehensive techno-environmental evaluation of an isolated PV/wind/biomass hybrid microgrid employing various battery technologies: A comparative analysis
Source: PLoS One. 2025 Apr 8;20(4):e0322420. doi: 10.1371/journal.pone.0322420 (PMC11978063; doi:10.1371/journal.pone.0322420)
Supplement: S2 File — (PDF) [file pone.0322420.s002.pdf]

PLOS ONE

RESEARCH ARTICLE

Comprehensive techno-environmental evaluation of an isolated PV/wind/biomass hybrid microgrid employing various battery technologies: A comparative analysis

Mohammed Alqahtani[1]*, Saeed Alhajri[2], Ahmed S. Menesy[2], Ali Maher Mohammed[2], Hamdy M. Sultan[3,4], Muhammad Khalid[2,5]*

1 Industrial Engineering Department, King Khalid University, Abha, Saudi Arabia, **2** Electrical Engineering Department, King Fahd University of Petroleum & Minerals (KFUPM), Dhahran, Saudi Arabia, **3** Faculty of Engineering, Electrical Engineering Department, Minia University, Minia, Egypt, **4** Faculty of Engineering, Department of Mechatronics Engineering, Nahda University in Beni Suef, Beni Suef, Egypt, **5** IRC for Sustainable Energy Systems (IRC-SES), KFUPM, Dhahran, Saudi Arabia

* m.alqahtani@kku.edu.sa (MA); mkhalid@kfupm.edu.sa (MK)

Check for updates

OPEN ACCESS

Citation: Alqahtani M, Alhajri S, S. Menesy A, Maher Mohammed A, M. Sultan H, Khalid M (2025) Comprehensive techno-environmental evaluation of an isolated PV/wind/biomass hybrid microgrid employing various battery technologies: A comparative analysis. PLoS ONE 20(2): e0317757. https://doi.org/10.1371/journal.pone.0317757

Editor: Chandan Kumar Shiva, SR University, INDIA

Received: August 17, 2024

Accepted: January 4, 2025

Published: February 20, 2025

Copyright: © 2025 Alqahtani et al. This is an open access article distributed under the terms of the Creative Commons Attribution License, which permits unrestricted use, distribution, and reproduction in any medium, provided the original author and source are credited.

Data Availability Statement: All relevant data are within the manuscript and its Supporting Information files.

Funding: The authors would like to express their profound gratitude to King Abdullah City for Atomic and Renewable Energy (K.A.CARE) for their financial support in accomplishing this work at KFUPM, and the author Mohammed Alqahtani also acknowledge the support by the Deanship of

Abstract

Renewable energy technologies offer promise for addressing energy access and environmental concerns, especially in remote off-grid areas. This paper presents a comprehensive techno-economic analysis of an off-grid PV/wind/biomass hybrid system. Employing optimization techniques including the osprey optimization algorithm (OOA), zebra optimization algorithm (ZOA), and flying foxes optimization (FFO) algorithm, the study aims to determine the optimal sizing of solar PV, wind, biomass, and battery components. Using data from Tabuk, Saudi Arabia (28.38˚ N, 36.56˚ E), the study seeks to achieve optimal sizing for solar PV, wind, biomass, and battery storage components to minimize the net present cost (NPC) and ensure reliable power supply, adhering to specified loss of power supply probability (LPSP) and excess energy thresholds. Three battery types, namely, flooded lead-acid, lithium iron phosphate (LFP), and nickel iron (Ni-Fe), were analyzed. Results reveal that ZOA outperformed other algorithms, supplying electricity at a minimum cost of 0.1285 $/kWh in one configuration, with the LFP battery achieving the lowest NPC of 3.8 M$ in case studies with constrained LPSP. Across multiple simulations, ZOA displayed superior stability and convergence characteristics, evidenced by its tight objective function range and lower relative error metrics. These findings underscore the potential of this integrated approach to enhance the economic viability and operational resilience of off-grid hybrid microgrid systems, particularly in arid and semi-arid regions.

1. Introduction

Over the past two decades, there has been a significant shift from dependence on fossil fuels to the adoption of renewable energy sources, driven by the need for cleaner, more sustainable

Scientific Research through King Khalid University, Saudi Arabia funded by the Large Group Research Project RGP2/392/45.

Competing interests: The authors declares no conflict of interest.

Abbreviations: PV, photovoltaic; PSO, particle swarm optimization; LCA, life cycle assessment; EMS, energy management system; WT, wind turbine; IEC, international electrotechnical committee; MSW, municipal solid waste; BESS, battery energy storage system; LFP, lithium ferro phosphate; Ni-Fe, nickel iron; NPC, net present cost; DOD, depth of discharge; O&M, operations and maintenance; SOC, state of charge; EC, energy cost; LPSP, loss of power supply probability; NPC, net present cost; CRF, capital recovery factor; LPS, loss of power supply; ZOA, zebra optimization algorithm; OOA, osprey optimization algorithm; FFO, flying foxes optimization; RE, relative error; MAE, mean absolute error; SD, standard deviation; **Symbol**, **Description**; H, height; S, speed; $S(t)$, wind speed; t, time; Scutin, cut-in wind speeds; Scutoff, cut-off wind speeds; β_{wt}, wind turbine friction coefficient; N_{wt}, number of wind turbines; η_{wt}, efficiency of the wind turbine; pratedwt, rated power of the wind turbine; η_g, efficiency of the syngas; P_{BG}, output power of the generator; F_{Cons_BG}, average fuel consumption per hour; LHV_{pg}, lower heat value of the product gas; m_{pg}, mass flow of the product gas; LHV_B, lower heat value of the biomass; N_{BG}, number of biomass generators employed; B_{rated}, biomass consumption rate per hour (kg/h); PG_{rated}, the rated power output of the biomass generator; $P_{re}(t)$, biomass system; η_{CH}, efficiency factors for battery charging; η_{DIS}, efficiency factors for battery discharging; σ, self-discharge rate; N_{pv}, number of pv panels; N_{wt}, number of wind turbines; N_{BG}, number of biomass gasifier units; N_B, number of batteries; Canntot, total annualized cost of all system components; Canncap, annual interest accrued on initial capital investment; Cannfuel, annual cost of fuel used by biomass gasifier units; $C_{O\&M}$, annual operation and maintenance costs; C_{rep}, annual replacement cost for each subsystem; P_{inv}, inverter power; $P_L(t)$, rated power of load demands; η_{inv}, efficiency of the inverter; X_i, i^{th} zebra within a population; $x_{i,j}$, value suggested by the ith zebra for the jth problem variable; N, total number of population members (zebras); m, indicates the number of decision variables; F, vector comprising the values of the objective function; F_i, the objective function value attained for the ith zebra; Xinew,P1, the updated status of the ith zebra; xi,jnew,P1, updated status of the jth dimensional value; Finew,P1, new objective function value of the updated status; PZ, pioneer

power [1–3]. Hybrid renewable energy systems integrating resources like solar, wind, and biomass, offer a compelling alternative for remote and rural areas where energy access remains a critical challenge [4]. While hydrogen shows promise as a long-term energy storage medium to increase renewables penetration into the grid, off-grid hybrid systems present an immediate solution for regions without grid access [5,6]. These systems provide a stable power supply, reduce environmental impacts, and lower operational costs, yet require careful optimization to maximize resource efficiency and ensure reliability at minimal cost. In the midst of the Arab region's renowned wealth of energy resources, a pressing reality persists: millions of households and vital economic sectors grapple with inadequate access to dependable energy services. Although the Arab region is known for its abundant energy resources, a stark reality persists: millions of households and vital economic sectors continue to grapple with inadequate access to dependable energy services. The challenge of accessing electricity persists due to the often-unreliable national power grids across many countries [7]. Consequently, many regions have turned to decentralized diesel-based technologies to meet the energy demands of both domestic and commercial sectors. Diesel engines serve a multitude of purposes, from lighting homes and powering industrial equipment to facilitating irrigation and pumping activities.

This energy insecurity stands as a formidable obstacle, hindering the path to equitable, sustainable, and enduring development in these nations. However, amidst these challenges, solar photovoltaic (PV) technologies emerge as a promising solution, offering a cost-effective means to reduce diesel consumption for electricity generation and water pumping in rural and remote areas [8,9]. Nevertheless, the intermittency of most renewable sources poses a considerable challenge, particularly in fluctuating climatic conditions, significantly impacting energy production [10].

To address this dilemma, a strategic blend of diverse renewable energy sources becomes imperative to ensure a stable and reliable energy supply. Building upon earlier research, recent efforts on off-grid hybrid renewable systems have underscored the potential of integrating multiple renewable technologies, such as PV panels, wind turbines, fuel cells, and biomass solutions [11–16]. These innovative systems have not only demonstrated proof-of-concept but have also showcased enhanced efficiency, paving the way for a more sustainable energy landscape in the region.

The integration of renewable energy sources into hybrid microgrid systems presents a promising avenue for addressing global energy demands sustainably. Recent advancements in optimization algorithms have significantly contributed to enhancing the efficiency and reliability of these systems. This literature review encapsulates recent studies focusing on optimization techniques, renewable energy integration, and technological advancements in battery storage, providing a comprehensive overview of current research directions and findings in the domain.

A techno-economic assessment and environmental impact analysis of hybrid storage systems integrated into microgrids are detailed by Ikram et al. [17]. Their research employs particle swarm optimization (PSO) to manage the energy dispatch strategy effectively, demonstrating the potential of meta-heuristic algorithms in optimizing microgrid operations. This aligns with the document's focus on employing various battery technologies to evaluate their impact on the system's net present cost and environmental footprint. Recent research introduced by Zhao and Zhang [18], highlights a groundbreaking strategy for urban microgrid management, focusing on energy efficiency and reliability through innovative optimization techniques. This study underscores the importance of addressing renewable energies' uncertainty, presenting a model that significantly contributes to the Net-Zero smart cities initiative. Similarly, Hamza et al. [19], explore the enhancement of local energy sharing within peer-to-peer prosumer communities using a hybrid algorithm combining cellular automata and deep

zebra, stands as the best member; PZ_j, the pioneer zebra j^{th} dimension; r, random number within the interval [0; 1]; Xinew,P2, the updated status of the jth zebra; xi,jnew,P2, the updated status of the jth dimensional value; Finew,P2, the new objective function value of the updated status; t, iteration count; T, maximum number of iterations; R, constant equal to 0.01; P_s, probability of randomly selecting one of two strategies; AZ, status of the zebra under attack; AZ_j, zebra under attack j^{th} dimensional value.

learning, showcasing the smart node's capabilities in optimizing microgrid communities. The importance of optimized energy storage in enhancing microgrid reliability is explored by Zhong et al. [20], who propose a bi-layer optimization method based on the Beluga Whale Algorithm. This research highlights the critical role of energy storage systems in maintaining the microgrid's topology structure and ensuring efficient energy management. The authors in [21], explored the use of microgrids to manage renewable energy resources, addressing technical, economic, and environmental challenges. It introduces a distributable resource management strategy (DRMS) for hybrid microgrids, aimed at reducing costs, energy loss, and emissions while meeting operational constraints. Using the grey wolf optimizer (GWO) to determine optimal component sizes, the strategy achieved reductions of 1.06% in total net cost, 8.69% in energy loss, and 17.19% in emissions, with results confirmed by the firefly algorithm (FA) and particle swarm optimization (PSO). Sensitivity analysis supports DRMS's effectiveness in improving hybrid microgrid performance across economic, technical, and environmental measures.

Ayed et al. [22], conducted a case study in Thala city, Tunisia, presenting an optimal design and techno-economic analysis of on-grid, hybrid renewable energy systems utilizing wind and biomass resources. This study employs advanced optimization techniques to determine the most cost-effective scale for a hybrid energy system, providing a valuable framework for assessing the feasibility and economic viability of similar projects in other rural areas. Liu et al. [23], explored a multi-objective optimization model based on life cycle assessment (LCA) for the optimal design of hybrid solar and biomass systems. This approach offers a comprehensive evaluation of the environmental and economic impacts of integrating solar and biomass energy, aligning closely with the development of a mathematical model for optimal sizing and ensuring efficient energy utilization within hybrid systems. Zaman [24], provided an intensive analysis of the energy management system (EMS) for hybrid electric vehicles powered by renewable energy sources, including solar, wind, and biomass. Although focused on vehicle applications, this research offers insights into the integration of diverse renewable sources and the optimization of energy distribution, which are relevant to the optimization techniques explored in the primary study. The authors in [25], focused on optimizing a hybrid biomass-PV microgrid for agricultural use in Egypt. The study presents a new hybrid algorithm (HIWO/PSO), which combines invasive weed optimization (IWO) and particle swarm optimization (PSO), to determine the optimal sizing of the energy system. The performance of this system is evaluated using three key metrics: total net present cost (TNPC), LPSP, and excess energy fraction (EEF). The conclusions suggest that the HIWO/PSO algorithm outperforms other methods like harmony search (HS), standalone PSO, and flower pollination algorithm (FPA), by achieving optimal results more quickly and with fewer iterations. The results highlight the economic, ecological, and social benefits of the optimized microgrid design, suggesting that it can meet current and future energy needs efficiently. Barakat et al. [26], evaluated the potential of an on-grid hybrid renewable energy (HRE) system in Egypt, combining solar, wind, and biomass energy sources. The study is motivated by feed-in tariffs promoting renewable energy and aims to assess both environmental and economic impacts of the proposed system. The optimization, sizing, and cost analysis were performed using HOMER software, focusing on a typical small village. The results indicate that the most cost-effective combination of solar, wind, and biomass energy comes primarily from the biomass generator due to the availability of resources at the location. This hybrid system significantly reduces costs and CO2 emissions compared to a grid-connected system, demonstrating that renewable energy sources are a viable solution to meet rural electricity demands in Egypt. Samy et al. [27], focused on the technical and economic analysis of a hybrid PV-Biomass microgrid designed to supply power to a remote apple farm in the Albaha region of Saudi Arabia. The goal was to

optimize the system's components, PV panels, biomass generators, and batteries, to minimize the total annual cost (TAC) and NPC. Two optimization algorithms, harmony search (HS) and firefly algorithm (FA), were used to find the best configuration of system components, ensuring reliable power supply while keeping the LPSP at 2%. The study's conclusions confirm the effectiveness of the hybrid system design in providing a sustainable power solution for the farm, highlighting the system's potential applicability in other locations with similar renewable resources. Sensitivity analysis was performed to assess how variables like interest rates, inflation, and biomass availability could affect the system's long-term economic viability. The study demonstrates that the optimized system is capable of meeting both current and future energy needs in a cost-effective, environmentally friendly, and socially beneficial way.

The authors in [28], focused on analyzing the feasibility of using renewable energy (solar, wind, and biomass) for powering a remote rural area in Al-Jouf, Saudi Arabia. Four hybrid system configurations, PV/Wind, PV/Biomass, Wind/Biomass, and PV/Wind/Biomass, were simulated and optimized using HOMER software. The goal was to identify the most cost-effective and energy-efficient system. The study evaluates each configuration based on key economic metrics, including the NPC and the LCOE. The results show that the PV/Biomass system was the most economical choice, with a total NPC of 138,521.40 $ and an LCOE of 0.099 $/kWh. While renewable energy systems are environmentally and economically advantageous, the study highlights the challenge of high upfront investment costs, especially for low-income communities. To overcome this barrier, the paper suggests the need for support from government and non-governmental organizations to finance such projects. Idris et al. [29], performed a techno-economic and emission analysis of biomass-integrated hybrid energy systems for automotive condition monitoring centers. Utilizing simulation software, this study estimated the biomass resource availability and its potential integration within hybrid systems, providing a direct comparison to the research paper's objective of evaluating biomass as a viable component in hybrid renewable energy systems. Modu et al. [30], introduced an enhanced Salp Swarm Algorithm for the optimal design of a grid-independent solar-fuel cell-biomass energy system. The research highlights the algorithm's effectiveness in optimizing the configuration of hybrid renewable energy systems, directly contributing to the comparative analysis of optimization techniques outlined in the primary study.

Presented in this study is the design of an efficient off-grid renewable energy system tailored for supplying electricity to a remote village situated in Saudi Arabia. The proposed system harnesses the abundant solar and biomass resources available within the village. To achieve optimal sizing of this system, a meta-heuristic optimization approach is employed, leveraging the strengths of three distinct optimization techniques. In this context, the meta-heuristic algorithm demonstrating the highest performance will be selected for implementation. The optimization methods utilized encompass the Osprey Optimization Algorithm, Zebra Optimization Algorithm, and Flying Foxes Optimization Algorithm. These specific algorithms are chosen for their proven track record in effectively managing large-scale searches, consistently and swiftly converging towards the global optimum. This comprehensive approach ensures a robust and efficient off-grid renewable energy solution tailored to the unique energy needs of the village in Saudi Arabia.

The design process meticulously determines the ideal capacities of various renewable energy sources, including solar photovoltaic (PV), wind, biomass, and battery banks. This selection process is centered on the objective of minimizing the net present cost associated with the units provided by the proposed system, while also considering a predetermined loss of power supply probability. By carefully calibrating the capacities of each renewable source, the system aims to achieve optimal efficiency and cost-effectiveness in meeting the energy needs of the remote village in Saudi Arabia.

The main contributions of this study can be highlighted as follow:

- Proposing an optimized hybrid PV/wind/biomass microgrid design tailored for remote areas, with a case study focused on a specific rural location in Saudi Arabia, emphasizing economic viability and reliable power supply.
- Developing a comprehensive mathematical model to determine the optimal sizing of hybrid system components, maximizing energy efficiency, cost-effectiveness, and reliability.
- Employing three advanced optimization techniques, namely, the osprey optimization algorithm, zebra optimization algorithm, and flying foxes optimization algorithm, to achieve an optimized configuration, enabling superior performance and stability in hybrid microgrid systems.
- Comparative analysis of the results obtained from the three optimization algorithms to identify the most effective solution.
- Assessing the techno-economic performance of flooded lead-acid (FLC), lithium ferro phosphate (LFP), and nickel iron (Ni-Fe) batteries.
- Conducting sensitivity analyses to verify the developed algorithms robustness in solving the studied optimization problem.

The subsequent sections of this paper are structured as follows: Section 2 delves into the problem formulation; Section 3 outlines the proposed optimization procedures in detail. Moving forward, Section 4 provides a comprehensive analysis of the simulation results and ensuing discussions. Finally, Section 5 encapsulates the findings and draws insightful conclusions based on the study's outcomes.

2. Problem formulation

2.1 Modeling of the proposed system

As depicted in Fig 1, the proposed hybrid power system comprises key components such as PV panels, a wind farm, a Biomass Gasifier with a syngas engine, a battery bank, a dump load, and an inverter. PV panels, wind farm, and Biomass generators are employed to fulfill the load demand, with any surplus energy exceeding the demand utilized to charge the battery banks when available. The energy stored within the battery banks is subsequently utilized to meet the load requirements during periods of low energy production by the PV, wind, and biomass systems. Additionally, a dump load, functioning as an electrical resistance heater, is integrated into the system to manage the full generated capacity, thereby preventing the battery from overcharging. This comprehensive setup ensures efficient utilization of renewable energy sources and effective management of energy fluctuations to sustain continuous power supply.

2.1.1 Solar photovoltaic system modelling. Modeling the PV system within hybrid energy systems involves a meticulous process to accurately represent its behavior and interactions with other renewable sources. The PV system model typically includes various components to simulate its performance under different conditions. The present study uses a simulation model to assess the performance of the PV module, with Eq (1) applied to determine the output power of the PV generator [31,32]:

$$P_{PV} = \left(P_{rated} N_{pv} A_m D_f\right)\left(\frac{G}{R_{ref}}\right)\left(1 + K_T\left(T_C T_{ref}\right)\right) \quad (1)$$

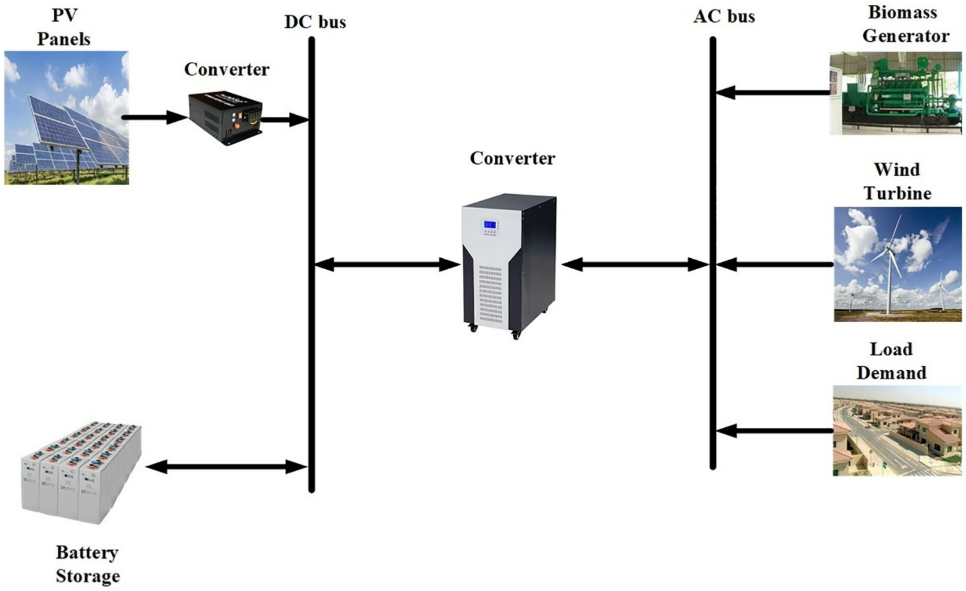

Fig 1. Schematic diagram of the studied off grid hybrid energy system.

https://doi.org/10.1371/journal.pone.0317757.g001

Here, $K_T = -3.7\times10^{-3}(1^{\circ}C)$ for monocrystalline and polycrystalline silicon. Additionally, based on the energy balance, it was suggested that T_C can be expressed as follows in Eq (2):

$$T_C = T_{amb} + G\left(\frac{NOCT - 20}{0.8}\right) \tag{2}$$

Where $NOCT = 43^{\circ}C$.

2.1.2 Wind system modelling. Wind energy stands out as a renewable energy source that operates without generating any greenhouse gas emissions. The wind turbine, comprising three fundamental components, namely the tower, the blades facilitating airflow for rotation, and the generator, plays a pivotal role in this energy conversion process [33]. When wind passes through the blades, part of its kinetic energy is transferred to the generator, where it is converted into electrical energy. The amount of electrical energy generated by a wind turbine (WT) depends on both the wind speed and the design of the blades. Consequently, wind turbines utilized for industrial electricity or lighting purposes are typically erected atop tall towers. The wind speed, which escalates with altitude above ground level, can be calculated at a specific height H_2 using the equation below [33,34]:

$$\left(\frac{S_2}{S_1}\right) = \left(\frac{H_2}{H_1}\right)^{\beta_{wt}} \tag{3}$$

Here, S_1 and S_2 represent the wind speed at reference point H_1 and at the turbine hub height H_2, respectively. β_{wt} denotes the WT friction coefficient, which, according to the International Electrotechnical Committee (IEC) for normal wind conditions and 0.11 for more intense wind conditions. The power generated by wind turbines (P_{wt}) can be expressed

through the following equation [31]:

$$P_{wt} = \begin{cases} 0 & S(t) < S_{cut}^{in} \text{ or } S(t) > S_{cut}^{off} \\ N_{wt} p_{rated}^{wt} \eta_{wt} \left(\frac{S^2(t) - S_{cut}^2}{S_{rated}^2 - S_{cut}^2} \right) & S_{cut}^{in} < S(t) < S_{rated} \\ N_{wt} p_{rated}^{wt} \eta_{wt} & S_{rated} < S(t) < S_{cut}^{off} \end{cases} \quad (4)$$

In this equation, $S(t)$ represents the wind speed at time t, while S_{cut}^{in} and S_{cut}^{off} denote the cut-in and cut-off wind speeds, respectively. Furthermore, N_{wt} stands for the number of wind turbines, η_{wt} represents the efficiency of the wind turbine, and p_{rated}^{wt} is the rated power of the wind turbine. This model serves to accurately predict the power generation capabilities of the wind turbine within the hybrid energy system, considering the varying wind speeds and operational conditions.

2.1.3 Biomass generator modelling. Biomass resources encompass a diverse array of natural and derived materials, including woody and herbaceous species, wood waste, bagasse, agricultural and industrial residues, waste paper, municipal solid waste (MSW), sawdust, biosolids, grass, food processing waste, animal byproducts, aquatic plants, and algae. These resources primarily consist of three major organic components: cellulose, hemicelluloses, and lignin. The majority of biomass energy production stems from wood and wood waste (comprising 64%), followed by MSW (24%), agricultural waste (5%), and landfill gases (5%). There are three primary methods for utilizing biomass: it can be combusted to generate heat and electricity, converted into gas-like fuels such as methane, hydrogen, and carbon monoxide, or transformed into liquid fuels.

Liquid fuels, known as biofuels, predominantly manifest as two forms of alcohol: ethanol and methanol. The direct conversion of biomass into liquid fuels holds promise as a substantial source for meeting transportation fuel demands for cars, trucks, buses, airplanes, and trains. This becomes particularly significant given that approximately one-third of our nation's energy is currently allocated to transportation needs. Within the Kingdom of Saudi Arabia (KSA), major biomass resources include municipal solid wastes (MSWs), agricultural crops and residues, and sewage (water-carried waste). The estimated potentials for biomass energy over the next 20 years, especially in electricity generation for the KSA, are outlined in Table 1.

In this investigation, we utilize agricultural crops and residues as the primary biomass input for the gasifier. Here, the biomass particles undergo decomposition at temperatures exceeding 700 degrees Celsius. This decomposition process unfolds in four distinct stages, facilitated by the gasification agent, which, in this case, is air. Through this process, the particles recombine to generate a synthetic gas known as syngas. This syngas serves as the fuel for turbines, which, in turn, generate electricity [35].

Table 1. Estimated Bio-energy potential from various biomass sources in the kingdom of Saudi Arabia for the years 2014 and 2034.

Biomass source	Million ton (estimated)	Bio-energy in 2014, mtoe	Bio-energy in 2034, mtoe
Municipal solid wastes	10.5	3.6	4.7
Agricultural crops and residues	3.5	1.2	1.7
Forestry crops and residues	0.5	0.2	0.3
Agro-industrial residues	0.8	0.3	0.5
Sewage (water-carried waste)	2.5	0.5	0.8
**Total biomass**	17.8	5.8	8

https://doi.org/10.1371/journal.pone.0317757.t001

The efficiency of the syngas, denoted as η_g, the output power of the generator, P_{BG}, and the average fuel consumption per hour, F_{Cons_BG}, are determined using the following equations [35,36]:

$$\eta_g = \frac{LHV_{pg}\, m_{pg}}{LHV_B\, m_B} \tag{5}$$

$$P_{BG}(t) = \frac{N_{BG}}{F_m}\left(\frac{\eta_g\, LHV_B\, B_{rated}}{LHV_{pg}} - F_0\, PG_{rated}\right) \tag{6}$$

$$F_{Cons\_BG}(t) = \frac{LHV_{pg}}{\eta_g\, LHV_B}\left(N_{BG}\, F_0\, PG_{rated} + F_m\, P_{BG}(t)\right) \tag{7}$$

Here, LHV_{pg} represents the lower heat value of the product gas, while m_{pg} signifies the mass flow of the product gas. Similarly, LHV_B denotes the lower heat value of the biomass, with m_B indicating the mass flow of biomass. The variable N_{BG} represents the number of biomass generators employed, and B_{rated} stands for the biomass consumption rate per hour (kg/h). Finally, PG_{rated} signifies the rated power output of the biomass generator.

2.1.4 Battery energy storage system (BESS). The battery serves as a crucial component for storing electrical energy in a chemical form, later releasing it as needed in the form of electrical power. Its primary function is to store excess power generated by PV units, wind turbines (WTs), and the biomass system when production surpasses current load demands. When renewable sources fall short of meeting demand, or during system maintenance, the batteries begin discharging stored energy.

This study includes a comparative analysis among the Flooded lead-acid, Lithium Ferro Phosphate (LFP), and Nickel Iron (Ni-Fe) Battery (Ni-Fe) batteries within the context of a PV/Biomass hybrid system. The objective of this comparison is to explore the impact of different battery types on the Net Present Cost (NPC) of the system. The lead-acid battery comprises distinct positive and negative electrodes, crafted from lead dioxide and metallic lead respectively. These electrodes are immersed within a diluted sulfuric acid electrolyte. Two common variants exist: the flooded lead-acid and sealed valve-regulated lead-acid (VRLA) solutions. Among these, the flooded lead-acid is the more economical option but necessitates regular maintenance, typically monthly, to monitor and replenish distilled water. Moreover, proper ventilation is crucial due to its emission of flammable gases. In this study, a 100 Ah, 4.8 kWh Flooded Lead Acid battery bank, comprising four batteries, each with 50% Depth of Discharge (DOD) and 0.5% self-discharge per day, was employed [30]. The associated costs include $672 for initial and replacement expenses, with $25 for Operations and Maintenance (O&M) costs, over a lifetime of 4 years at an efficiency of 85%.

The Li-ion battery represents an advanced rechargeable alternative. During charging, Li + ions are extracted from the cathode oxide compound and integrated into the anode lattice. The cathode boasts high potential yet a poor Li state, while the anode exhibits low potential but a high Li state [30]. Li-ion batteries offer numerous advantages over their counterparts, including high energy density (attributed to their elevated output voltage), exceptional efficiency, extended cycle life, and environmental sustainability.

The Lithium Ferro Phosphate (LFP) battery, introduced in 1997, marked a significant reduction in lithium battery costs, facilitating widespread commercial usage. Distinguished by its thermal and cycling stability, safety features, and environmental robustness, the LFP battery stands out as one of the most promising Li-ion batteries for grid integration. In this context, a

102.4 Ah, 5.2 kWh LFP battery bank with 70% DOD and 0.033% self-discharge per day was employed [30]. The associated costs include an initial and replacement expense of $5,890, O&M costs of $2, all over a battery lifetime of 15 years at an efficiency of 95%.

The Nickel-Iron (Ni-Fe) battery, comprising nickel (III) oxide-hydroxide positive plates and iron-based negative plates, operates with a potassium hydroxide electrolyte. Known for its robustness in challenging environments, the Ni-Fe battery boasts an extended lifespan, rendering it ideal for renewable energy backup applications. For this battery type, a 100 Ah, 4.8 kWh bank with 80% DOD and 1% self-discharge per day was utilized [30]. The associated costs include $3,880 for initial and replacement expenses, $5 for Operations and Maintenance (O&M) costs, all over a battery lifetime of 25 years at an efficiency of 96%.

The input power into the battery can be either positive or negative, depending on the battery's state of charge (SOC_{Bat}), indicating whether it is in a charging or discharging state. The SOC_{Bat} values during charging (B_{CH}) and discharging (B_{DIS}) modes are calculated using the following equations [37]:

$$B_{CH}(t) = min[(P_{re}(t) - P_L(t)) * \eta_{CH}] \tag{8}$$

$$P_{re} = P_{pv} + \frac{P_{wt} + P_{BG}}{\eta_{inv}} \tag{9}$$

$$SOC_{Bat}(t) = SOC_{Bat}(t-1) * (1-\sigma) + B_{CH}(t) \tag{10}$$

$$B_{DIS}(t) = min[(P_L(t) - P_{re}(t)) * \eta_{DIS}] \tag{11}$$

$$SOC_{Bat}(t) = SOC_{Bat}(t-1) * (1-\sigma) - B_{DIS}(t)(t) \tag{12}$$

The equation involves the calculation of the total hourly power generated by the combined PV, WT, and biomass system, denoted as $P_{re}(t)$. The efficiency factors for battery charging (η_{CH}) and discharging (η_{DIS}) play a crucial role, alongside the self-discharge rate represented by σ.

2.2 Objective function structure and system constraints

The goal of optimizing this hybrid system is to achieve the most reliable and cost-effective operation. This is accomplished by minimizing two factors: Energy Cost (EC) and Loss of Power Supply Probability (LPSP). Additionally, the power absorbed by the dummy load is considered. To achieve this optimal balance, four continuous decision variables need to be adjusted:

N_{pv}: Number of photovoltaic (PV) panels

N_{wt}: Number of wind turbines

N_{BG}: Number of biomass gasifier units

N_B: Number of batteries

The following equations represent the objective function along with the established constraints.

$$OF = \min F(x) = \min(\alpha_1 COE + \alpha_2 LPSP + \alpha_3 EXE) \tag{13}$$

$$LPSP = \sum_{1}^{T} \frac{LPS(t)}{P_L(t)} \tag{14}$$

$$LPS(t) = P_L(t) - (P_{re}(t) + SOC_{Bat}(t-1) - SOC_{min}) * \eta_{inv} \tag{15}$$

$$EXE = \sum\nolimits_{1}^{T} \frac{P_d(t)}{P_L(t)} \tag{16}$$

$$X = [N_{pv}\ N_{wt}\ N_{BG}\ N_B] \tag{17}$$

$$N_{pv}^{min} \le N_{pv} \le N_{pv}^{max} \tag{18}$$

$$N_{wt}^{min} \le N_{wt} \le N_{wt}^{max} \tag{19}$$

$$N_{BG}^{min} \le N_{BG} \le N_{BG}^{max} \tag{20}$$

$$\mathrm{N_B^{min}} \le \mathrm{N_B} \le \mathrm{N_B^{max}} \tag{21}$$

$$LSPS \le LSPS_{max} \tag{22}$$

The weights, α_1,α_2, and α_3, are determined through a trial-and-error process to achieve the best possible outcome. In this study, their values are set to 0.3, 0.5, and 0.2, respectively, with the sum restricted to be less than 1.

2.3 Cost of Energy (COE)

This section explains how to calculate three key costs associated with the hybrid system: total annual cost ($\mathrm{C_{ann}^{tot}}$), energy cost (COE), and net present cost (NPC). Our primary goal is to optimize the system for reliable power delivery at the lowest possible overall cost, COE.

$$NPC = \frac{C_{ann}^{tot}}{CRF} \tag{23}$$

$$C_{ann}^{tot} = C_{ann}^{cap} + C_{ann}^{fuel} + \sum_{i=1}^{S} C_{\mathrm{O\&M},i} + \sum_{i=1}^{S} C_{rep,i} \tag{24}$$

$$CRF = \frac{r(I+1)^S}{(r+1)^S - 1} \tag{25}$$

$$COE = \frac{C_{ann}^{tot}}{P_L^{tot}} \tag{26}$$

The total annual cost (C_{ann}^{tot}) considers various expenses associated with the hybrid system:
C_{ann}^{tot}: Total annualized cost of all system components.
C_{ann}^{cap}: Annual interest accrued on the initial capital investment.
C_{ann}^{fuel}: Annual cost of fuel used by the biomass gasifier units.

$C_{O\&M}$: Annual operation and maintenance costs for each subsystem within the hybrid system.

C_{rep}: Annual replacement cost for each subsystem, considering their lifespan.

To calculate the annualized costs, a Capital Recovery Factor (CRF) is used. This factor considers the interest rate (r = 6%) and the system's lifespan (S = 25 years). Additionally, P_L^{tot} represents the total energy demand of the system throughout a year (8760 hours).

2.4 Energy management strategy

This study explores a hybrid energy system that relies primarily on renewable sources like solar panels (PV), wind turbines, and biomass gasification. Battery banks act as a backup to ensure uninterrupted power flow and system reliability. The power management strategy focuses on utilizing renewable energy and inverter power to meet the designated load demands, where $P_{inv} = P_L(t)/\eta_{inv}$.

Fig 2 offers a clear view of the flowchart outlining the proposed system's operating strategy.

- When renewable generation capacity perfectly matches the load demand (t), there's no change in battery storage (unchanged capacity). Consequently, the Loss of Power Supply (LPS) for this time period is zero (LPS(t) = 0)
- If renewable energy generation exceeds the load demand (t) and the battery isn't fully charged, the excess power will be used to charge the battery.
- Upon reaching its maximum charge state ($SOC_{Bat}(t) = SOC_{Bat}(t-1)$), the battery ceases further energy intake. To prevent the hybrid system from exceeding its output capacity, any additional power is diverted to a dedicated "dummy load" for dissipation.
- In instances where renewable generation falls short of the load demand (t) and the battery state of charge exceeds the minimum threshold (if $P_{re}(t) < P_{inv}(t)$ and $SOC_{Bat}(t-1)^*(1-\sigma) > SOC_{min}$), the battery storage system will discharge to bridge the gap and satisfy the load requirement.

3. Proposed methodology

3.1 Zebra optimization algorithm (ZOA)

Zebras, originating from eastern and southern Africa, are equine animals renowned for their striking black-and-white striped coats. These stripes, typically running vertically along their necks and bodies, serve as effective camouflage against predators and also help fend off biting flies. The physical specifications of zebras are as follows: they measure between 210–300 cm in body length, possess tails ranging from 38–75 cm, stand at shoulder heights of 110–160 cm, and weigh anywhere from 175–450 kg. These animals are built for strength and agility, equipped with long, slender legs that allow them to achieve impressive speeds when needed [38].

Similar to other wild equines, zebras have a single toe on each foot, a long neck, and a head that aids in grazing on ground-level grass. Within their social structures, two key behaviors stand out: foraging and defense against predators. During foraging, a lead zebra paves the way for the herd, guiding them to grazing grounds. In the face of danger, zebras employ a zigzag escape pattern to evade predators. Occasionally, they also gather together to confuse or intimidate the threat. These behaviors underscore the adaptability and survival strategies of these remarkable animals in their natural habitat.

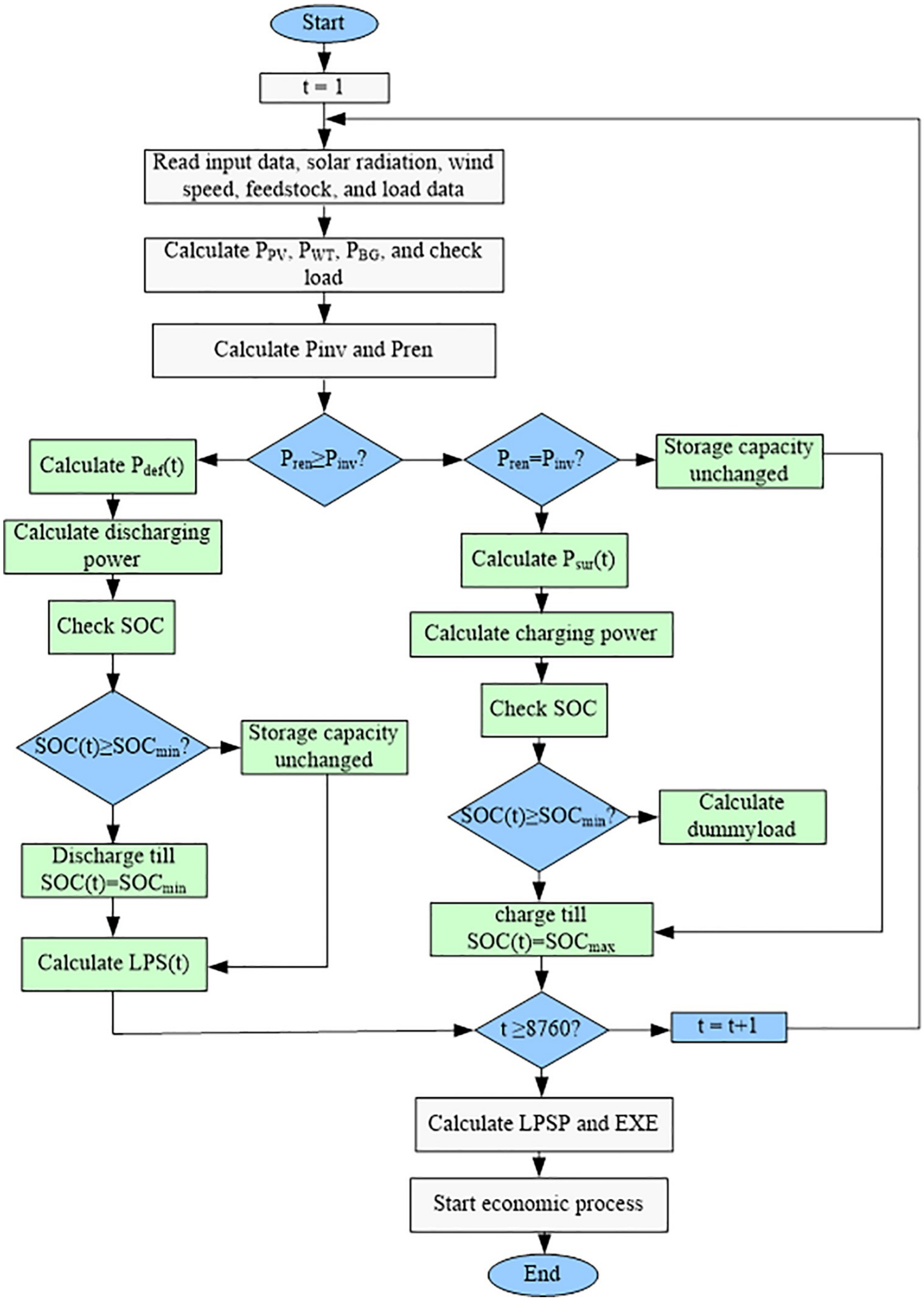

Fig 2. Energy management strategy of the proposed system.

https://doi.org/10.1371/journal.pone.0317757.g002

1) Initialization. ZOA functions as a population-based optimizer with zebras comprising its population. Mathematically, each zebra signifies a candidate solution within the search space, navigating across the landscape of potential solutions to explore the optimization problem's variables. The position of each zebra within this search space dictates the values assigned to the decision variables. Consequently, each zebra, as a member of ZOA, can be conceptualized using a vector, where the elements of this vector correspond to the problem variables'

values. The collective group of zebras can be mathematically depicted through a matrix. Initially, the zebras' positions within the search space are randomly allocated. The specifics of the ZOA population matrix are detailed in Eq (27) [38].

$$X = \begin{bmatrix} X_1 \\ \vdots \\ X_i \\ \vdots \\ X_N \end{bmatrix} = \begin{bmatrix} x_{1,1} & \cdots & x_{1,j} & \cdots & x_{1,m} \\ \vdots & \ddots & \vdots & \ddots & \vdots \\ x_{i,1} & \cdots & x_{i,j} & \cdots & x_{i,m} \\ \vdots & \ddots & \vdots & \ddots & \vdots \\ x_{N,1} & \cdots & x_{N,j} & \cdots & x_{N,m} \end{bmatrix} \tag{27}$$

In this context, let X denote the zebra population, where X_i represents the i-th zebra within this population. The value suggested by the ith zebra for the jth problem variable is denoted as $x_{i,j}$. Here, N stands for the total number of population members (zebras), while m indicates the number of decision variables. Each zebra represents a potential solution to the optimization problem, allowing the objective function to be evaluated based on the specific values proposed by each zebra for the problem's variables. The resulting values from this assessment are delineated as a vector using Eq (28).

$$X = \begin{bmatrix} F_1 \\ \vdots \\ F_i \\ \vdots \\ F_N \end{bmatrix} = \begin{bmatrix} F(X_1) \\ \vdots \\ F(X_i) \\ \vdots \\ F(X_N) \end{bmatrix} \tag{28}$$

Here, F denotes the vector comprising the values of the objective function, with F_i representing the objective function value attained for the i^{-th} zebra. The comparison of these objective function values serves as an effective method to analyze the quality of their respective candidate solutions and pinpoint the optimal solution for the given problem.

In scenarios involving minimization, the zebra with the lowest objective function value emerges as the ideal candidate solution. Conversely, in cases of maximization, the zebra with the highest objective function value stands out as the most optimal candidate. Since the positions of the zebras, and subsequently the values of the objective function, are updated in each iteration, it becomes imperative to identify the best candidate solution within each iteration as well. Two innate behaviors observed in wild zebras have been utilized to update members of ZOA. These behaviors encompass: (i) Foraging, and (ii) Defensive strategies against predators.

2) Phase 1: Foraging behavior. During the initial phase, adjustments are made to the population members based on simulations that mimic zebra behavior during their search for food. Zebras predominantly subsist on grasses and sedges, yet in instances of scarcity in their favored food sources, they may turn to buds, fruits, bark, roots, and leaves for sustenance. The amount of time zebras dedicate to feeding, ranging from 60–80 percent, is contingent upon the quality and availability of vegetation.

Among the zebras, there exists a specific type known as the plains zebra, renowned as a trailblazing grazer. By consuming the less nutritious upper canopy of grasses, this zebra creates favorable conditions for other species that thrive on shorter, more nourishing grasses below. Within the ZOA framework, the top-performing member of the population is likened to this

pioneer zebra, guiding other population members towards its position within the search space. Consequently, the process of updating the zebras' positions during the foraging phase can be mathematically represented using Eqs (29) and (30) [38].

$$x_{i,j}^{new,P1} = x_{i,j} + r.(PZ_j - I.x_{i,j}) \tag{29}$$

$$X_i = \begin{cases} X_i^{new,P1} & F_i^{new,P1} < F_i \\ X_i & else \end{cases} \tag{30}$$

In the first phase, where $X_i^{new,P1}$ represents the updated status of the i-th zebra, $x_{i,j}^{new,P1}$ denotes its jth dimensional value, and $F_i^{new,P1}$ is its new objective function value. *PZ* refers to the pioneer zebra, which stands as the best member, with PZ_j representing its j-th dimension. Here, *r* symbolizes a random number within the interval [0; 1]. Additionally, $I\epsilon[1,2]$ and if the parameter *I* equals 2, there will be significantly more alterations in the movement of the population.

3) Phase 2: Defensive strategies against predators. In the second phase of the ZOA, simulations are conducted to mimic the defense strategies of zebras against various predators. The primary predators of zebras include lions, cheetahs, leopards, wild dogs, brown hyenas, spotted hyenas, and crocodiles near water sources. Zebras' defensive responses differ based on the predator they encounter. When facing lion attacks, zebras employ a zigzag escape pattern and random sideways movements. Against smaller predators like hyenas and wild dogs, zebras exhibit a more aggressive approach, aiming to confuse and intimidate the attackers by grouping together. In the ZOA design, it assumes that one of two conditions occurs with equal probability: either (i) the zebra is attacked by a lion, prompting an escape strategy, or (ii) other predators attack, leading to an offensive response.

The first strategy, for lion attacks, involves zebras escaping from the immediate vicinity of the attack, mathematically represented by mode S1 File in Eq (5). In the second strategy, when other predators target a zebra, the herd members rally to the attacked zebra's defense, creating a defensive formation to frighten and confuse the predator. This defensive strategy is mathematically described by mode S2 in Eq (31). When updating the positions of zebras, a new position is accepted if it improves the objective function's value at that location. This updating condition is expressed in Eq (32) [38].

$$x_{i,j}^{new,P2} = \begin{cases} S_1 : x_{i,j} + R.(2r-1).\left(1 - \frac{t}{T}\right).x_{i,j} & P_s \le 0.5, \\ S_2 : x_{i,j} + r.(AZ_j - I.x_{i,j}) & else \end{cases} \tag{31}$$

$$X_i = \begin{cases} X_i^{new,P2} & F_i^{new,P2} < F_i \\ X_i & else \end{cases} \tag{32}$$

Here, $X_i^{new,P2}$ represents the updated status of the i-th zebra following the second phase, with $x_{i,j}^{new,P2}$ denoting its jth dimensional value, and $F_i^{new,P2}$ indicating its new objective function value. The variable *t* signifies the iteration count, while *T* stands for the maximum number of iterations. *R* is a constant equal to 0.01, and P_S represents the probability of randomly selecting one of two strategies within the interval [0; 1]. Additionally, *AZ* denotes the status of the zebra under attack, with AZ_j representing its jth dimensional value. The steps of the Zebra Optimization Algorithm are illustrated in flowcharts in Fig 3.

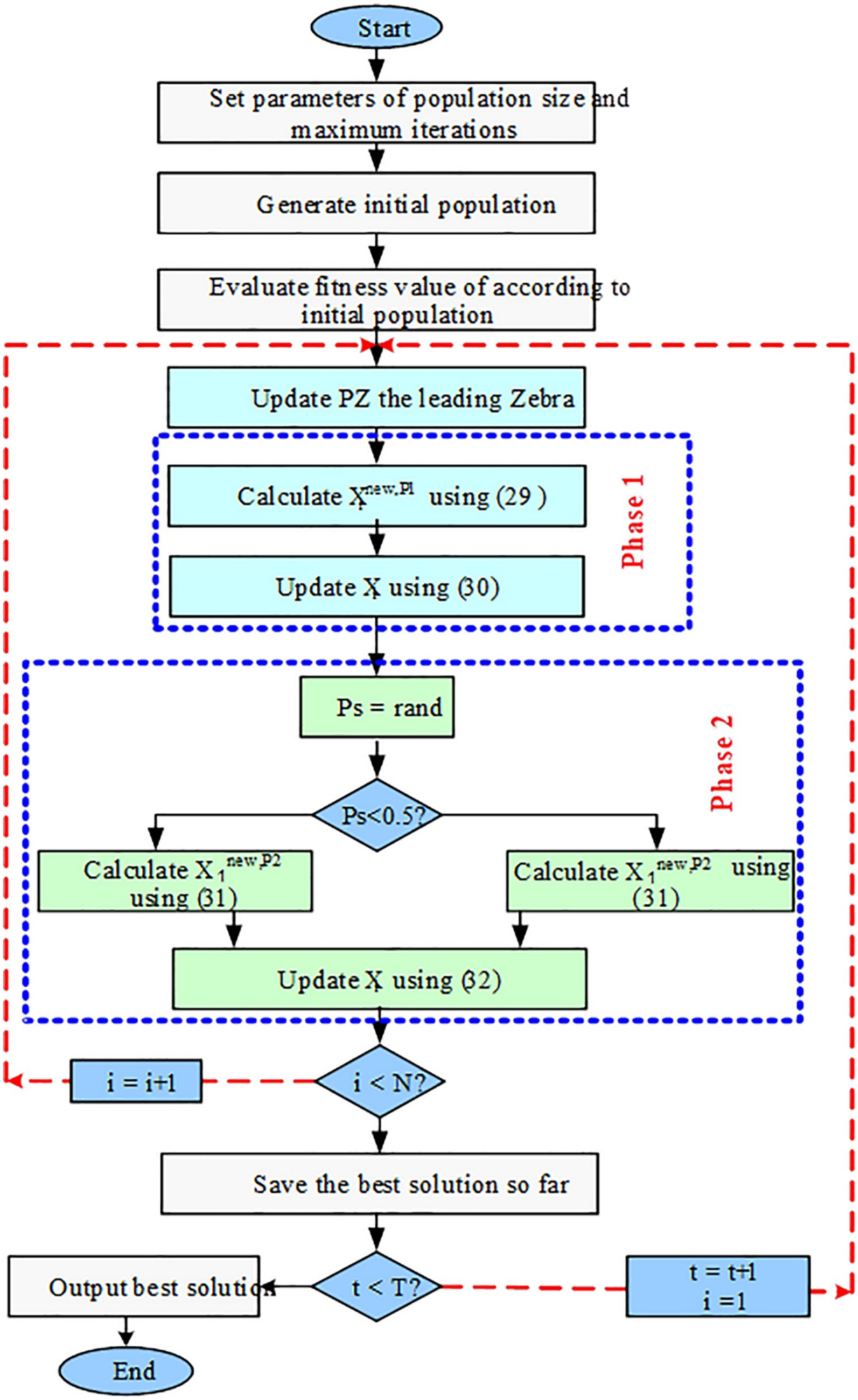

Fig 3. Flowchart of ZOA.

https://doi.org/10.1371/journal.pone.0317757.g003

3.2 Osprey optimization algorithm (OOA)

The osprey, also known as the fish hawk, river hawk, and sea hawk, is a diurnal bird of prey known for its cosmopolitan range and fish-eating habits. Typically measuring 50–66 cm in length, weighing 0.9–2.1 kg, and boasting a wingspan of 127–180 cm, the osprey is depicted in Fig 4. Its distinctive features include:

➢; Deep-glossy brown upperparts, with a white breast that may exhibit streaks of brown, and pure white underparts.

➢; A white head adorned with a black mask extending across the eyes to the sides of the neck.

➢; Golden to brown irises and a pale blue, transparent nictitating membrane.

➢; White feet with black talons and a black bill complemented by a blue cere.

➢; The osprey is recognized for its long, narrow wings and a short tail.

This piscivorous bird's diet primarily consists of fish, comprising around 99% of its food intake. Typically, it captures live fish ranging from 150 to 300 grams and 25–35 centimeters in length, though it can handle fish anywhere from 50 grams to 2 kilograms. Ospreys possess exceptional vision, aiding them in spotting underwater prey. Flying at heights of 10–40 meters above the water's surface, they locate fish beneath the water's surface, then swiftly descend, dipping their feet in the water before diving to secure their catch. Once captured, the osprey carries its prey to a nearby perch, usually a rock, where it begins to consume it. These intelligent behaviors of hunting and positioning for consumption serve as the foundation for a potential new optimization algorithm. The proposed OOA approach integrates mathematical models based on these osprey behaviors, discussed in detail in the following sections.

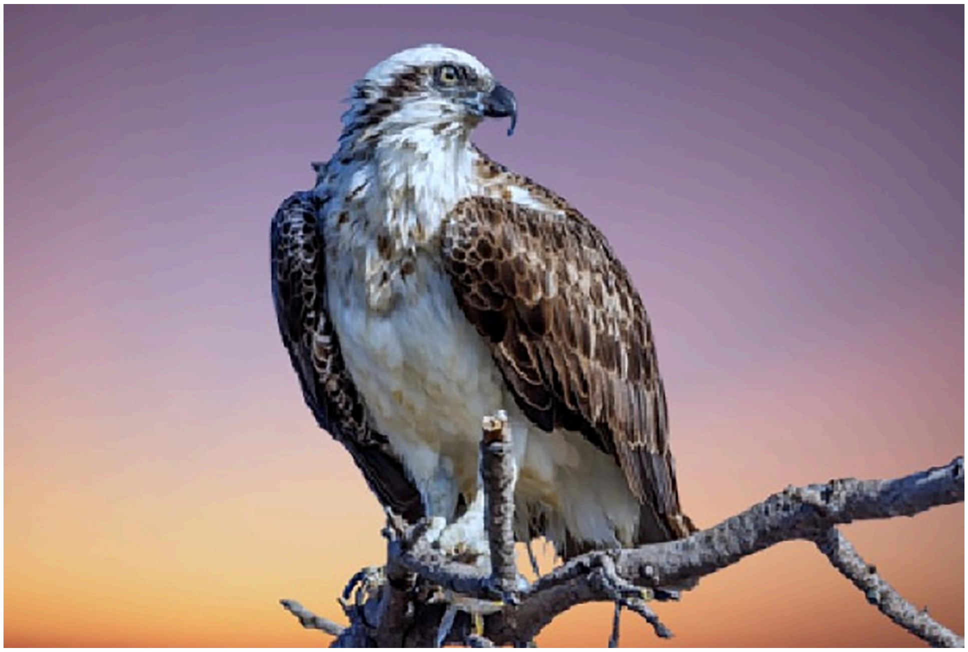

Fig 4. Osprey bird.

https://doi.org/10.1371/journal.pone.0317757.g004

1) Initialization. The proposed Optimization Osprey Algorithm (OOA) operates as a population-based method that seeks optimal solutions by leveraging the collective search capabilities of its members within the problem-solving domain through iterative processes. In this framework, each osprey within the OOA population defines values for problem variables based on its specific location within the search space. Consequently, each osprey represents a potential solution to the problem, structured mathematically as a vector. The collective of ospreys constitutes the OOA population, which can be represented as a matrix, as described in Eq (33). Initially, during the commencement of OOA execution, the positions of ospreys within the search space are randomly set using Eq (34).

$$X = \begin{bmatrix} X_1 \\ \vdots \\ X_i \\ \vdots \\ X_N \end{bmatrix} = \begin{bmatrix} x_{1,1} & \cdots & x_{1,j} & \cdots & x_{1,m} \\ \vdots & \ddots & \vdots & \ddots & \vdots \\ x_{i,1} & \cdots & x_{i,j} & \cdots & x_{i,m} \\ \vdots & \ddots & \vdots & \ddots & \vdots \\ x_{N,1} & \cdots & x_{N,j} & \cdots & x_{N,m} \end{bmatrix} \tag{33}$$

$$x_{i,j} = lb_j + r_{i,j} \cdot (ub_j - lb_j), \; i = 1, 2, \ldots, N, \quad j = 1, 2, \ldots., m \tag{34}$$

Here, X represents the matrix of ospreys' positions within the population, where X_i denotes the ith osprey (a potential solution), $x_{i,j}$ signifies its jth dimension (a problem variable), N stands for the total number of ospreys, and m represents the number of problem variables. The values $r_{i,j}$ are random numbers falling within the range [0, 1], while lb_j and ub_j indicate the lower and upper bounds, respectively, for the j-th problem variable.

Since each osprey serves as a potential solution to the problem, the corresponding objective function can be assessed for each osprey. The resulting evaluations of the problem's objective function can be illustrated as a vector, as demonstrated in Eq (35).

$$F = \begin{bmatrix} F_1 \\ \vdots \\ F_i \\ \vdots \\ F_N \end{bmatrix} = \begin{bmatrix} F(X_1) \\ \vdots \\ F(X_i) \\ \vdots \\ F(X_N) \end{bmatrix} \tag{35}$$

Here, F represents the vector containing the values of the objective function, where F_i denotes the specific objective function value obtained for the i-th osprey.

Phase 1: Position identification and hunting the fish

Ospreys exhibit remarkable hunting abilities, using their strong eyesight to detect fish locations underwater. Once they pinpoint the fish, ospreys dive underwater to capture their prey. The initial phase of population updating in OOA is inspired by this natural behavior of ospreys. Simulating the osprey's hunting technique results in substantial adjustments to the osprey's position within the search space, thereby enhancing OOA's exploration capabilities to pinpoint optimal regions and evade local optima.

In the OOA design, each osprey considers the positions of other ospreys in the search space with superior objective function values as underwater prey. The collection of potential prey for

each osprey is defined using Eq (36) [39].

$$FP_i = \{X_k | k \in \{1, 2, \ldots, N\} \bigwedge F_k < F_i\} \cup \{X_{best}\} \quad (36)$$

where FP_i is the set of fish positions for the i th osprey and X_{best} is the best candidate solution (the best osprey). The osprey randomly detects the position of one of these fish and attacks it. Based on the simulation of the movement of the osprey towardsthe fish, a new position for the corresponding osprey is calculated using (37). This new position, if it improves the value of the objective function, replaces the previous position of the osprey according to (38) [39].

$$x_{i,j}^{P1} = x_{i,j} + r_{i,j}.(SF_{i,j} - I_{i,j}.x_{i,j})$$

$$x_{i,j}^{P1} = \begin{cases} x_{i,j}^{P1} & lb_j \leq x_{i,j}^{P1} \leq ub_j \\ lb_j & x_{i,j}^{P1} < lb_j \\ ub_j & x_{i,j}^{P1} > ub_j \end{cases} \quad (37)$$

$$X_i = \begin{cases} X_i^{P1} & F_i^{P1} < F_{i;} \\ X_i & else, \end{cases} \quad (38)$$

Here, X_i^{P1} denotes the updated position of the i-th osprey following the first phase of the OOA. The term xP1i,j represents its jth dimension, while F_i^{P1} indicates its corresponding objective function value. SF_i refers to the fish selected by the ith osprey, with $SF_{i,j}$ representing its jth dimension. Additionally, $r_{i,j}$ represents random numbers within the range [0, 1], and $I_{i,j}$ are random numbers selected from the set [1,2].

Phase 2: Carrying the fish to the suitable position

After successfully capturing a fish, the osprey transports it to a secure and suitable location for consumption. The second phase of population updating in OOA is inspired by this natural behavior of ospreys. By simulating the act of carrying the fish to a safe spot, minor adjustments are made to the osprey's position within the search space. This adjustment enhances OOA's exploitation power, aiding in local search refinement and convergence towards improved solutions near the identified ones.

In the OOA design, to emulate this osprey behavior, a new random position is initially computed for each population member, designated as a 'suitable fish-eating position,' as outlined in Eq (39). Subsequently, if this new position yields an enhanced objective function value, it supplants the previous position of the corresponding osprey, as illustrated in Eq (40). This process mirrors the osprey's natural instinct, contributing to the algorithm's effectiveness in refining solutions and converging towards optimal outcomes [39].

$$x_{i,j}^{P2} = x_{i,j} + \frac{lb_j + r.(ub_j - lb_j)}{t}, i = 1, 2, \ldots .N, j = 1, 2, \ldots m, t = 1, 2, \ldots T \quad (39)$$

$$x_{i,j}^{P2} = \begin{cases} x_{i,j}^{P2} & lb_j \leq x_{i,j}^{P2} \leq ub_j \\ lb_j & x_{i,j}^{P2} < lb_j \\ ub_j & x_{i,j}^{P2} > ub_j \end{cases}$$

$$X_i = \begin{cases} X_i^{P2} & F_i^{P2} < F_{i;} \\ X_i & else, \end{cases} \tag{40}$$

Here, X_i^{P2} represents the updated position of the i-th osprey following the second phase of OOA. The term $x_{i,j}^{P2}$ denotes its jth dimension, while F_i^{P2} indicates its corresponding objective function value. The values of $r_{i,j}$ are randomly selected from the interval [0, 1]. Additionally, t serves as the iteration counter of the algorithm, and T signifies the total number of iterations. The procedural steps for implementing OOA are illustrated in the flowchart depicted in Fig 5.

3.3 Flying foxes optimization (FFO)

Flying foxes, among the world's largest bat species, rely primarily on their keen eyesight for navigation as they lack echolocation abilities. Unfortunately, many flying fox species are currently facing the threat of extinction, and these remarkable creatures encounter substantial challenges, particularly during heatwaves, which significantly disrupt their daily routines. Throughout the night, flying foxes diligently search their surroundings for food and return to their roosts come morning. As the daytime temperatures soar, they employ their wings as a protective shield against the heat. This behavior persists until they become fatigued and drained by noon. At this point, the flying foxes grapple with dehydration and struggle to breathe [40]. In their parched and distressed state, the flying foxes actively seek out cooler trees to find relief. During this time, they may either follow one another or independently search for a suitable tree. Unfortunately, a common occurrence is that the first bats to locate a cooler tree are swiftly overwhelmed and suffocated by the influx of numerous other bats desperately seeking respite. This tragic scenario results in the suffocation and loss of a significant number of flying foxes [40].

The Flying Foxes Optimization (FFO) is a stochastic algorithm rooted in the survival strategies employed by flying foxes in coping with elevated temperatures. FFO utilizes a hybrid algorithmic structure, amalgamating operators from established algorithms. The effectiveness of FFO is notably influenced by factors such as the population size (N), the replacement list (RL), and the attraction constant (b). Flying foxes are among the largest bat species, and their spatial navigation relies on their keen environmental awareness, as they lack echolocation abilities. Following their nightly feeding, they return to their habitat trees. Flying foxes actively search for cooler trees to perch on, seeking refuge from the increasing morning heatwaves. Unfortunately, in many cases, those that are the first to discover a sufficiently cool tree often become overwhelmed by other flying foxes and meet a tragic fate due to suffocation.

This innovative algorithmic approach commences by assembling an initial collection of positions derived from various flying foxes. These positions are represented as a vector, denoted as x = (x1,. . ., xm), featuring elements in a multi-dimensional space (m). Subsequently, the objective function is applied to assess these positions, reflecting the flying foxes' quest to find cooler trees to ensure their survival amid elevated temperatures. This optimization process emulates the natural behavior of flying foxes as they seek refuge in favorable environments. The movement patterns of flying foxes involve a process where they either follow

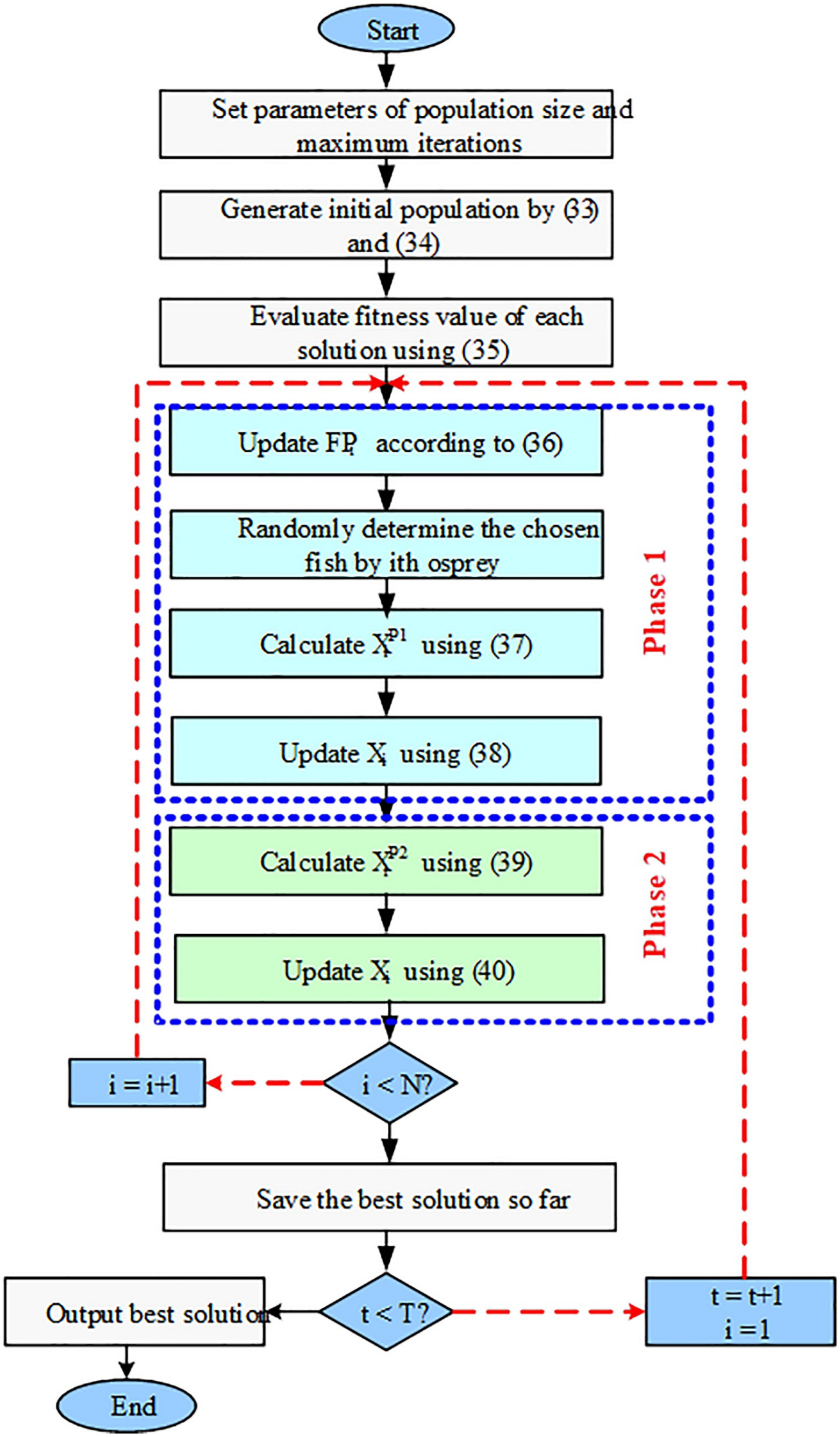

Fig 5. Flowchart of OOA.

https://doi.org/10.1371/journal.pone.0317757.g005

the paths of their peers or actively seek out the closest available tree when their current habitat tree fails to provide a suitable minimum temperature to escape the intense heat. This movement can be mathematically described by the following equation [40]:

$$x_{i,j}^{t+1} = x_{i,j}^{t} + a.rand(cool_j - x_{i,j}^{t}) \tag{41}$$

$x_i^0 \sim U(x_{min}, x_{max})$, the specific components of $FF(i)$ denoted as $x_{i,j}^t$ during iteration t, a constant value a, a random factor $rand \sim U(0,1)$, and the location of the flying fox situated in the tree with the lowest temperature, referred to as "cool." Eq (18) comes into play when the difference between the temperature at "cool" and the temperature at xi, represented by $|f(cool) - f(x_i)|$, exceeds a certain threshold $\frac{\delta_1}{2}$. In this context, "cool" represents the position of the flying fox in the coolest spot ever identified, which corresponds to the best solution found thus far. The parameter δ_1 defines the maximum distance within which two flying foxes are considered to be in proximity to each other.

When a flying fox approaches a tree with the lowest temperature and the difference in temperatures $|f(cool) - f(x_i)|$ is less than or equal to $\frac{\delta_1}{2}$, it seeks out the nearest available space to prevent overcrowding. Subsequent equations will provide further clarification on this behavior [40].

$$nx_{i,j}^{t+1} = x_{i,j}^{t} + rand_{1,j}(cool_j - x_{i,j}^{t}) + rand_{2,j}(x_{R1j}^{t} - x_{R2j}^{t}) \tag{42}$$

$$x_{i,j}^{t+1} = \begin{cases} nx_{i,j}^{t+1} & if\ j = k\ or\ rnd_j \geq Pa \\ x_{i,j}^{t} & otherwise \end{cases} \tag{43}$$

In this context, $rand \sim U(0,1)$ represents a random number uniformly distributed between 0 and 1, and rnd_j is another arbitrary number within the same range. The variables x_{R1j}^t and x_{R2j}^t refer to two random members selected from the current population, while Pa represents a constant probability. Lastly, the variable k is chosen randomly from the set {1,2,. . .,m}, ensuring that at least one element from $nx_{i,j}^{t+1}$ is incorporated into $x_{i,j}^{t+1}$, thus preventing any duplication between the original and new solutions.

A thorough evaluation of these computed solutions takes place. If a flying fox discovers the tree with the lowest temperature during this process, it is considered a new solution. Conversely, if it doesn't find the coolest tree, it returns to its previous location.

The demise of flying foxes can be attributed to various factors. For example, they might find themselves in a highly remote area with extreme heat while searching for the coolest tree, making it impossible for them to survive. In such situations, an alternative approach involves utilizing a Replacement List (RL) containing the unique optimal solutions from the NL (number of unique optimal solutions) available. An arbitrary integer, denoted as n and falling within the range of 2 to NL, is generated. The equation below illustrates how the position of a newly-created flying fox can be determined [40]:

$$x_{i,j}^{t+1} = \frac{\sum_{k=1}^{n} RL_{k,j}^{t}}{n} \tag{44}$$

where RL_k^t is the k-th flying fox on the RL during t reiteration. Eq (44) is geared towards maximizing the possibility of spotting an adequate region.

Flying foxes may also face mortality due to overcrowding by fellow population members. In such instances, a probability is calculated before concluding an iteration, taking into account the number of flying foxes located in the regions with the lowest temperature. This probability

can be represented as follows [40]:

$$pD = \frac{nc - 1}{population\ size} \tag{45}$$

Here, nc is closely tied to the count of flying foxes having objective functions similar to the optimal solution. Genetic crossover is employed to enable the mating of two flying foxes. The first step is to randomly choose two parents from the population, ensuring they are distinct. The crossover process results in the creation of two offspring, and this generation occurs as follows [40]:

$$offspring\ 1 = L.R_1 + (1 - L).R_2$$

$$offspring\ 2 = L.R_2 + (1 - L).R_2 \tag{46}$$

R_1 and R_2 are unique members of the population chosen at random, and L is a randomly generated value within the range of 0 to 1.

4. Simulation results and discussion

The technical and economic specifications of the proposed subsystems included in the isolated hybrid microgrid is presented in Table 2. The monthly average biomass materials available for utilization in the proposed area is demonstrated in the graph presented by Fig 6. While the annual solar irradiation, wind speed, average air temperature, and load demand in the proposed region are visualized in Figs 7–10 respectively.

The optimization algorithms were executed using the Matlab software, with specific parameters set for each algorithm:

Zebra Optimization Algorithm (ZOA): Number of search agents = 10, Maximum number of iterations = 50, dimension size = 4.

Osprey Optimization Algorithm (OOA): Search Agents = 10, Maximum number of iterations = 50.

Fly Foxes Optimization (FFO): Search Agents = 10, Maximum number of iterations = 50.

Throughout this research, three optimal configurations of sustainable hybrid systems were explored, each employing different battery energy storage devices:

Case (1): Flooded Lead Acid

Case (2): Lithium Ferro Phosphate (LFP)

Case (3): Nickel Iron Battery (Ni-Fe)

To enhance the reliability and robustness of the proposed algorithms and mitigate the impact of randomness, the optimization program utilizing ZOA, OOA, and FFO techniques was executed 50 times. The minimum value of the objective function was recorded for each run. Each algorithm was consistently applied across all three case studies.

The convergence behavior of the proposed hybrid cases using the ZOA, OOA, and FFO optimization algorithms is depicted in Fig 11 for 50 simulation runs. It is evident that the ZOA algorithm achieves final objective function values within a narrow range, signifying its stability in solving the optimization problem. For a more nuanced comparison, a comprehensive statistical analysis was conducted on the objective function values obtained from the 50 runs for all three cases. This analysis encompasses both parametric and nonparametric measures. The parametric measures include the minimum, maximum, and mean values of the objective function, while the nonparametric measures include the median, relative error (RE), mean absolute

Table 2. Technical and economic characteristics of the system components.

	Value	Unit
**PV System**		
cost	14854	$\$/m^2$
Temperature coefficient	0.0037	-
Efficiency	15	%
NOCT	25	˚C
Standard radiation	1000	W/m^2
lifetime	20	year
Replacement cost	13885	$
**WT System**		
Rated power	30	kw
Hub height	50	m
Efficiency	80	%
Cut in speed	2.5	m/s
Rated speed	12	m/s
Cut-off speed	25	m/s
lifetime	20	year
Cost	3200	$/kw
**Inverter**		
Efficiency	95	%
lifetime	10	year
cost	711	$/kw
Replacement cost	650	$/kw
**Biomass Generator**		
LHV_B	14.8	MJ/kg
LHV_{pg}	4.766	MJ/kg
B_{rated}	72	kg/h
η_g	80	%
PG_{rated}	40	KW
(F_0)	0.0644	kg/h/50kW
(F_m)	0.2998	kg/h/50kW
(BG_{cost})	16000	$/kw
lifetime	20 to 30	years

https://doi.org/10.1371/journal.pone.0317757.t002

error (MAE), standard deviation (SD), and efficiency. Efficiency, in this context, refers to the ratio between the minimum objective function value and the mean value. Table 3 summarizes the statistical results for ZOA, OOA, and FFO across the three cases. Fig 12 visually presents the distribution of the objective function values over the 50 runs, corroborating the findings in Table 3. Based on the comprehensive analysis, it can be concluded that the proposed ZOA algorithm exhibits superior performance compared to the OOA algorithm. This conclusion is supported by the tighter convergence range of ZOA in Fig 12, as well as its consistently lower objective function values across various statistical measures in Table 3.

The convergence curves for the best run within the 50 executions for all algorithms are presented in Fig 13. The results of the optimization process from the three proposed algorithms for case 1 are tabulated in Table 4. It is proved that the LPSP lies within the predefined constraints. The ZOA algorithm successes to supply the load with electricity at a rate of 0.128516381 $/kWh which is the minimum with respect to other algorithms. The distribution of the annual cost of the system with respect to the types of technology is tabulated in Table 5

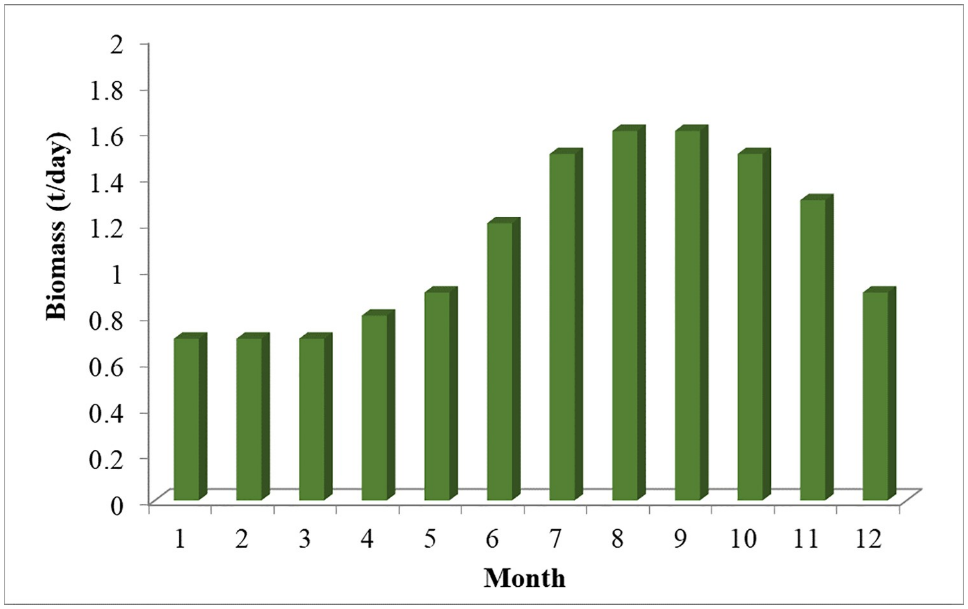

Fig 6. Available monthly biomass in the proposed area.

https://doi.org/10.1371/journal.pone.0317757.g006

and is presented graphically in the chart presented in Fig 14. Also, the division of the annual cost of each subsystem according to the type of cost is summarized in Table 6 and presented graphically in Fig 15.

Similarly, the convergence behavior of the proposed hybrid system in case 2, using the ZOA, OOA, and FFO optimization algorithms, is depicted in Fig 16 for 50 simulation runs. Table 7 summarizes the statistical results for ZOA, OOA, and FFO across the three cases. Fig 17 visually presents the distribution of the objective function values over the 50 runs, corroborating the findings in Table 7. Based on the comprehensive analysis, it can be concluded that the proposed ZOA algorithm exhibits superior performance compared to the OOA algorithm.

The convergence curves for the best run within the 50 executions for all algorithms are presented in Fig 18. The results of the optimization process from the three proposed algorithms

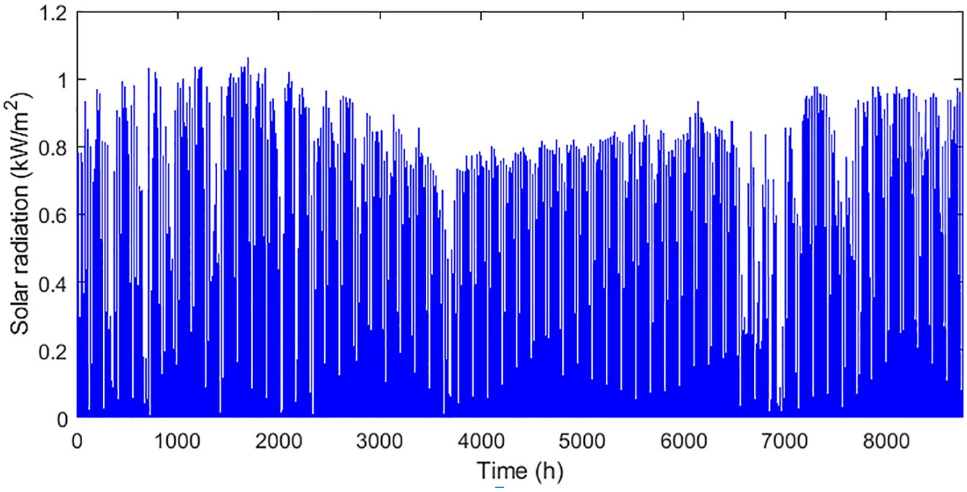

Fig 7. Available solar radiation in the proposed area.

https://doi.org/10.1371/journal.pone.0317757.g007

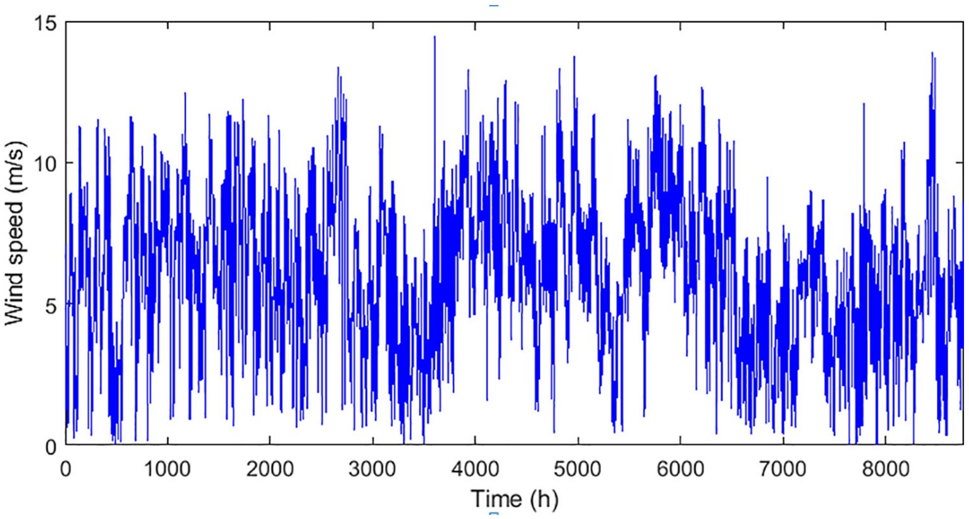

Fig 8. Available wind speed in the proposed area.

https://doi.org/10.1371/journal.pone.0317757.g008

for case 2 are tabulated in Table 8. It is proved that the LPSP lies within the predefined constraints. The ZOA algorithm successes to supply the load with electricity at a rate of 0.206554621 $/kWh which is the minimum with respect to other algorithms. The distribution of the annual cost of the system with respect to the types of technology is tabulated in Table 9 and is presented graphically in the chart presented in Fig 19. Also, the division of the annual cost of each subsystem according to the type of cost is summarized in Table 10 and presented graphically in Fig 20.

For case 3, the convergence behavior of the proposed hybrid system, using the ZOA, OOA, and FFO optimization algorithms, is depicted in Fig 21 for 50 simulation runs. Table 11 summarizes the statistical results for ZOA, OOA, and FFO across the three cases. Fig 22 visually presents the distribution of the objective function values over the 50 runs, corroborating the findings in Table 11. Based on the comprehensive analysis, it can be concluded that the proposed ZOA algorithm exhibits superior performance compared to the OOA algorithm.

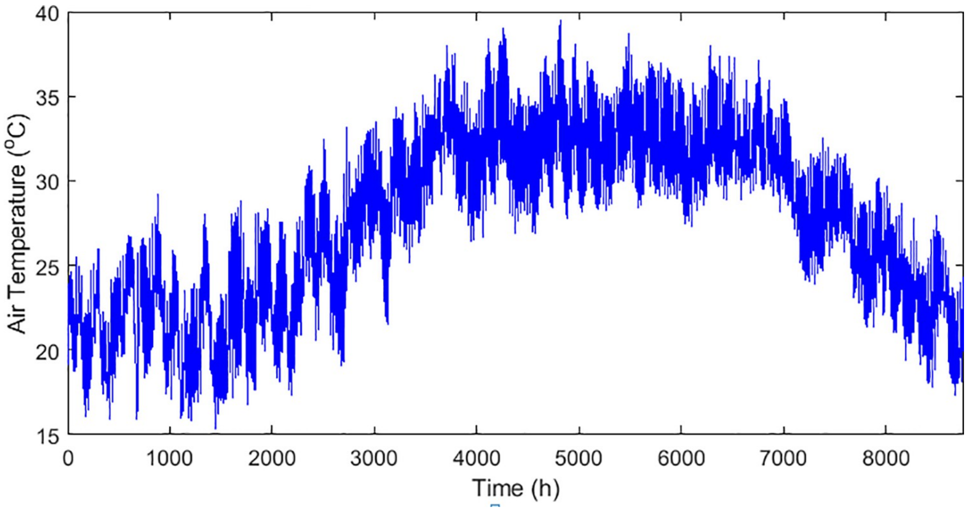

Fig 9. Average daily air temperature in the proposed area.

https://doi.org/10.1371/journal.pone.0317757.g009

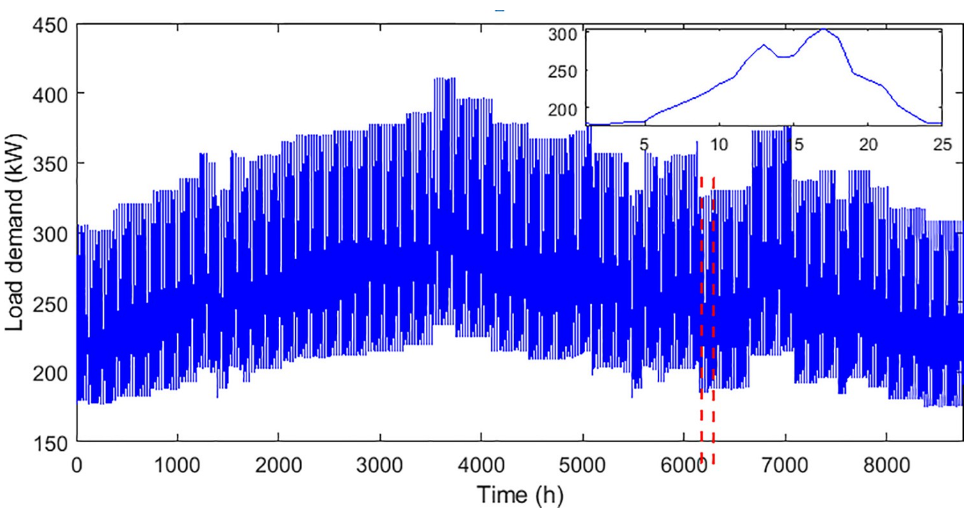

Fig 10. Annual load demand in the proposed area.

https://doi.org/10.1371/journal.pone.0317757.g010

The convergence curves for the best run within the 50 executions for all algorithms are presented in Fig 23. The results of the optimization process from the three proposed algorithms for case 3 are tabulated in Table 12. It is proved that the LPSP lies within the predefined constraints. The ZOA algorithm successes to supply the load with electricity at a rate of 0.15215951 $/kWh which is the minimum with respect to other algorithms. The distribution of the annual cost of the system with respect to the types of technology is tabulated in Table 13 and is presented graphically in the chart presented in Fig 24. Also, the division of the annual cost of each subsystem according to the type of cost is summarized in Table 14 and presented graphically in Fig 25.

Fig 26 displays a comprehensive time-series analysis of the power output from an isolated PV/Wind/Biomass hybrid microgrid utilizing Nickel Iron batteries, charted over a 24-hour cycle. This graph presents the temporal dynamics of power generation from photovoltaic, and biomass generators, alongside the power load, charging power, and discharging power. The plot reveals significant variability in power generation, with the photovoltaic output peaking during daylight hours and the wind turbine output showing higher variability, potentially influenced by diurnal and nocturnal wind patterns. Biomass energy contributes a relatively steady output, underscoring its role in stabilizing the hybrid system by compensating for the intermittency of solar and wind resources. Notably, the charging and discharging cycles of the battery are well-aligned with the demand-supply dynamics, indicating efficient energy storage management. This Fig highlights the critical interplay between different renewable sources and storage systems in maintaining energy balance and reliability in an off-grid setting, showcasing the effectiveness of the employed optimization strategy in enhancing the system's operational efficiency and sustainability.

Fig 27 depicts the State of Charge of the battery system for case 3 over an extensive duration of one year (8760 hours), effectively capturing the battery's charging and discharging cycles within the hybrid PV/Wind/Biomass microgrid. This graph provides a granular view of the SOC fluctuations, which is crucial for assessing the battery's performance and the overall efficiency of energy management within the system. it is clearly noticed that the SOC values oscillate between 20% and nearly 100%, reflecting a dynamic and responsive energy storage system that adapts to varying energy inputs from the renewable sources and the load demands. The frequent peaks near 100% indicate periods where the battery reaches full charge, likely

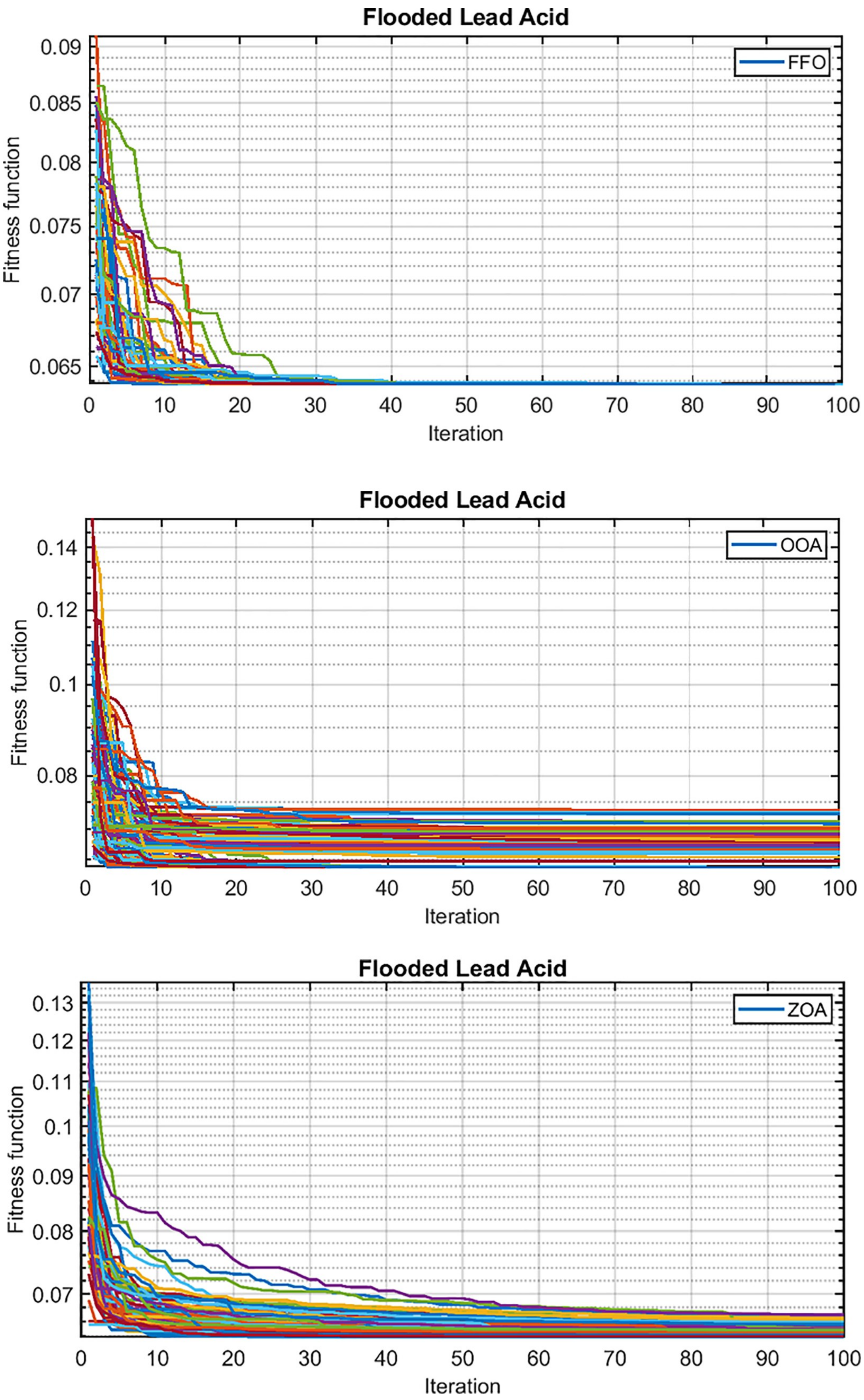

Fig 11. Fitness function over the 50 runs. (A) FFO. (B) OOA. (C) ZOA.

https://doi.org/10.1371/journal.pone.0317757.g011

Table 3. Results of the statistical analysis for case 1.

Statistical Metric	FFO	ZOA	OOA
**best**	0.0638860	0.06387603	0.06483645
**worst**	0.111596	0.06696692	0.07352600
**mean**	0.067171	0.06491671	0.06889674
**median**	0.065756	0.06471514	0.06894199
**SD**	0.673232	0.08250858	0.21116339
**RE**	2.57697	0.80665558	3.13118408
**RMSE**	0.007433	0.00131509	0.00456682

https://doi.org/10.1371/journal.pone.0317757.t003

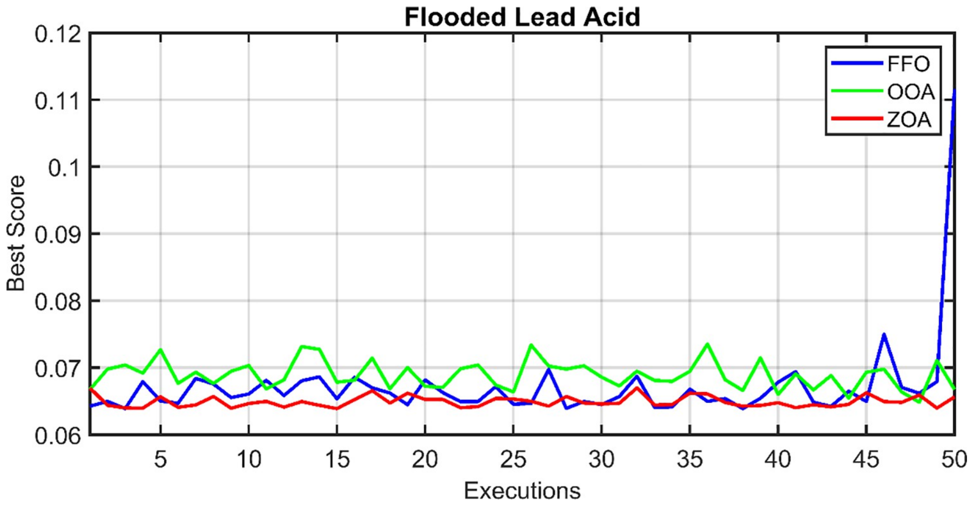

Fig 12. Final values of the convergence curves over the 50 runs.

https://doi.org/10.1371/journal.pone.0317757.g012

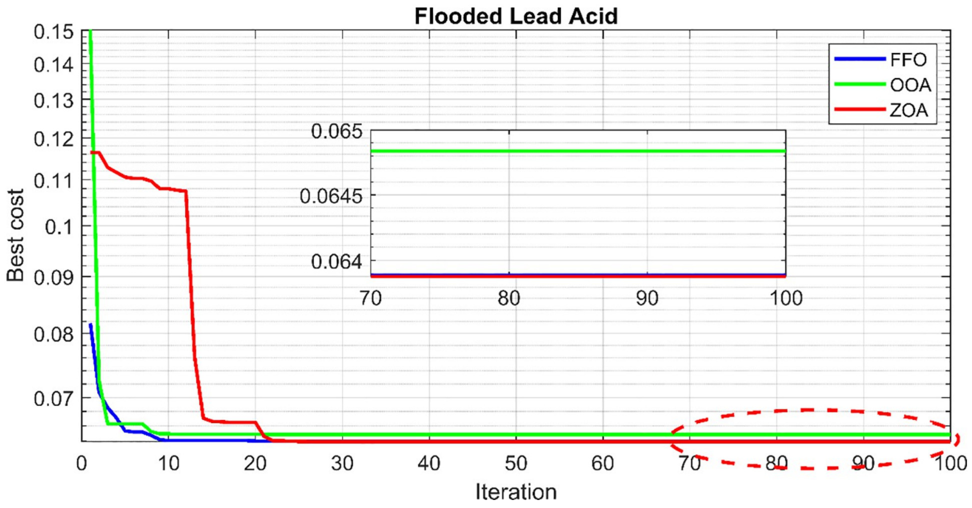

Fig 13. Convergence curves for case 1.

https://doi.org/10.1371/journal.pone.0317757.g013

Table 4. Optimization results of ZOA, OOA, and FFO for case 1.

Used Algorithm		FFO	ZOA	OOA
Best fitness		0.0638860	0.06387603	0.06483645
Optimized parameters	N_{PV}	21	19	14
	N_{WT}	1	2	13
	N_{BG}	2	2	2
	N_{batt}	601	600	596
COE		0.130737506	0.129817066	0.128516381
NPC		3829362.771	3802402.639	3764304.965
LPSP		0.004608407	0.003988321	0.005607505

https://doi.org/10.1371/journal.pone.0317757.t004

Table 5. Annual cost distributed between system components for case 1.

	PV	WT	BG	Battery	Inverter
FFO	$38315.95008	$833.3673428	$60668.42535	$131310.2277	$ 66321.509
OOA	$27532.65274	$8194.057251	$61912.90294	$130508.1015	$ 66321.509
ZOA	$41020.71109	$657.7756306	$60050.00187	$131508.4845	$ 66321.509

https://doi.org/10.1371/journal.pone.0317757.t005

corresponding to times of excess generation from photovoltaic and wind sources during favorable conditions. Conversely, the drops below 40% may signal higher energy consumption periods or less favorable conditions for energy generation. This visualization serves as an essential tool for understanding the robustness of the storage system in buffering against the intermittency of renewable energy sources and meeting load demands consistently. The rapid fluctuations also highlight the necessity for efficient battery management systems to optimize charging strategies and prolong battery life, ensuring sustainable and reliable system operation. This Fig underscores the critical role of advanced battery technologies and sophisticated management strategies in enhancing the viability of renewable off-grid systems.

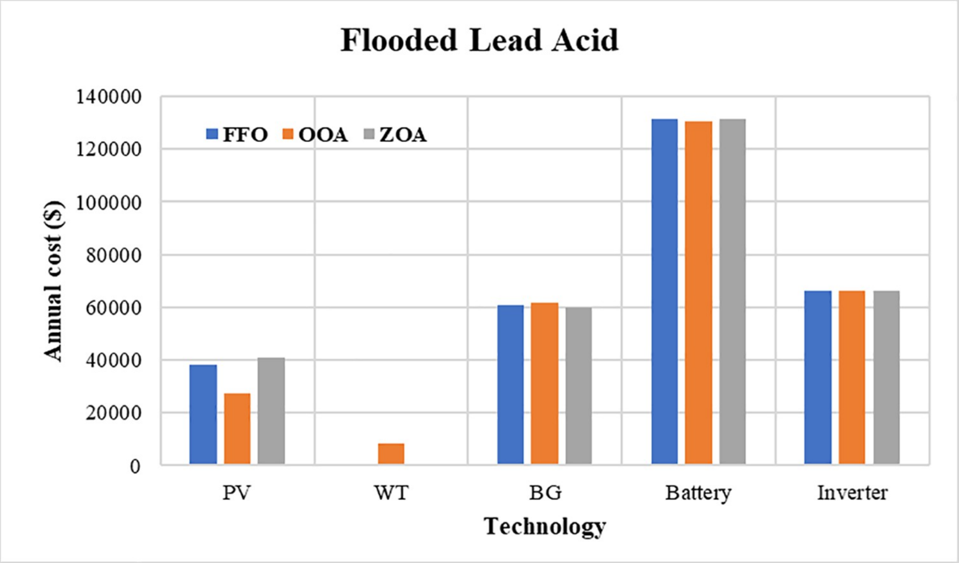

Fig 14. Annual cost divided between different subsystems for case 1.

https://doi.org/10.1371/journal.pone.0317757.g014

Table 6. Different types of cost distributed between system components for case 1.

	Used Algorithm	Capital Cost ($)	O&M Cost ($)	Replacement Cost ($)	Fuel Cost ($)
**PV**	FFO	24819.15081	191.647842	13305.15143	0
	OOA	17834.2716	137.7121922	9560.668942	0
	ZOA	26571.15934	205.1764537	14244.3753	0
**WT**	FFO	427.1507776	129.6435846	276.5729805	0
	OOA	4199.946107	1274.716323	2719.394822	0
	ZOA	337.149487	102.3274926	218.2986509	0
**BG**	FFO	2400.094919	19655.95445	0	38612.37598
	OOA	2449.32752	20059.15257	0	39404.42286
	ZOA	2375.629556	19455.59151	0	38218.7808
**Battery**	FFO	116315.9214	14994.30625	0	0
	OOA	115605.39	14902.71152	0	0
	ZOA	116491.5393	15016.94519	0	0
**Inverter**	FFO	39645.50935	0	26676	0
	OOA	39645.50935	0	26676	0
	ZOA	39645.50935	0	26676	0

https://doi.org/10.1371/journal.pone.0317757.t006

Fig 28 provides an insightful graphical representation of the hourly variation of load demand, surplus, and deficit power over a one-week period for case 3 using a Nickel Iron battery in the hybrid PV/Wind/Biomass system. This Fig effectively highlights the dynamic interplay between production and consumption within the hybrid system, demonstrating the critical role of the Nickel Iron battery in managing fluctuations in energy availability and demand. Notably, the ability to handle these disparities with minimal deficit occurrences suggests a well-optimized system that can potentially offer stable and reliable power supply in off-grid applications. This visualization is crucial for evaluating the system's efficiency, the effectiveness of the storage solution, and the potential need for further system adjustments or capacity enhancements to better match the load demand profiles.

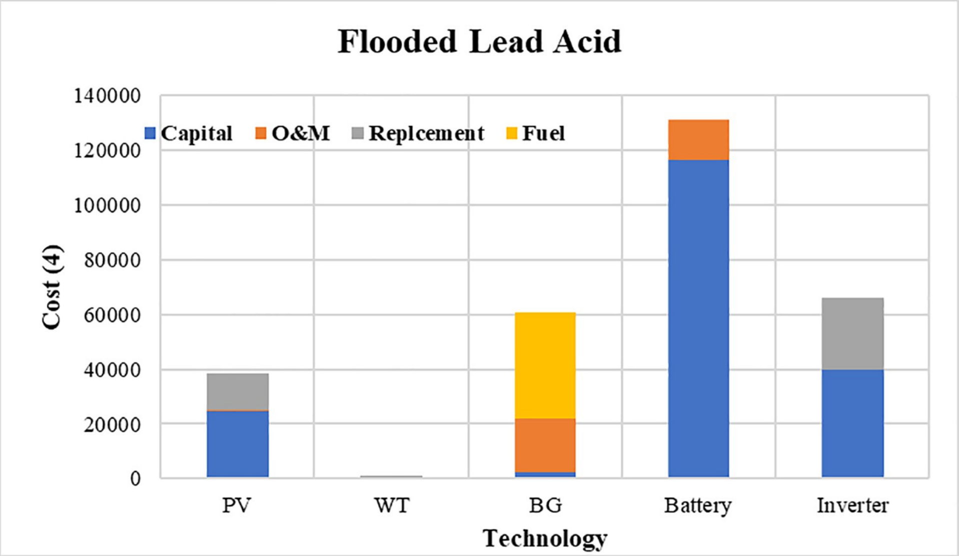

Fig 15. Division of annual cost with respect to cost type for case 1.

https://doi.org/10.1371/journal.pone.0317757.g015

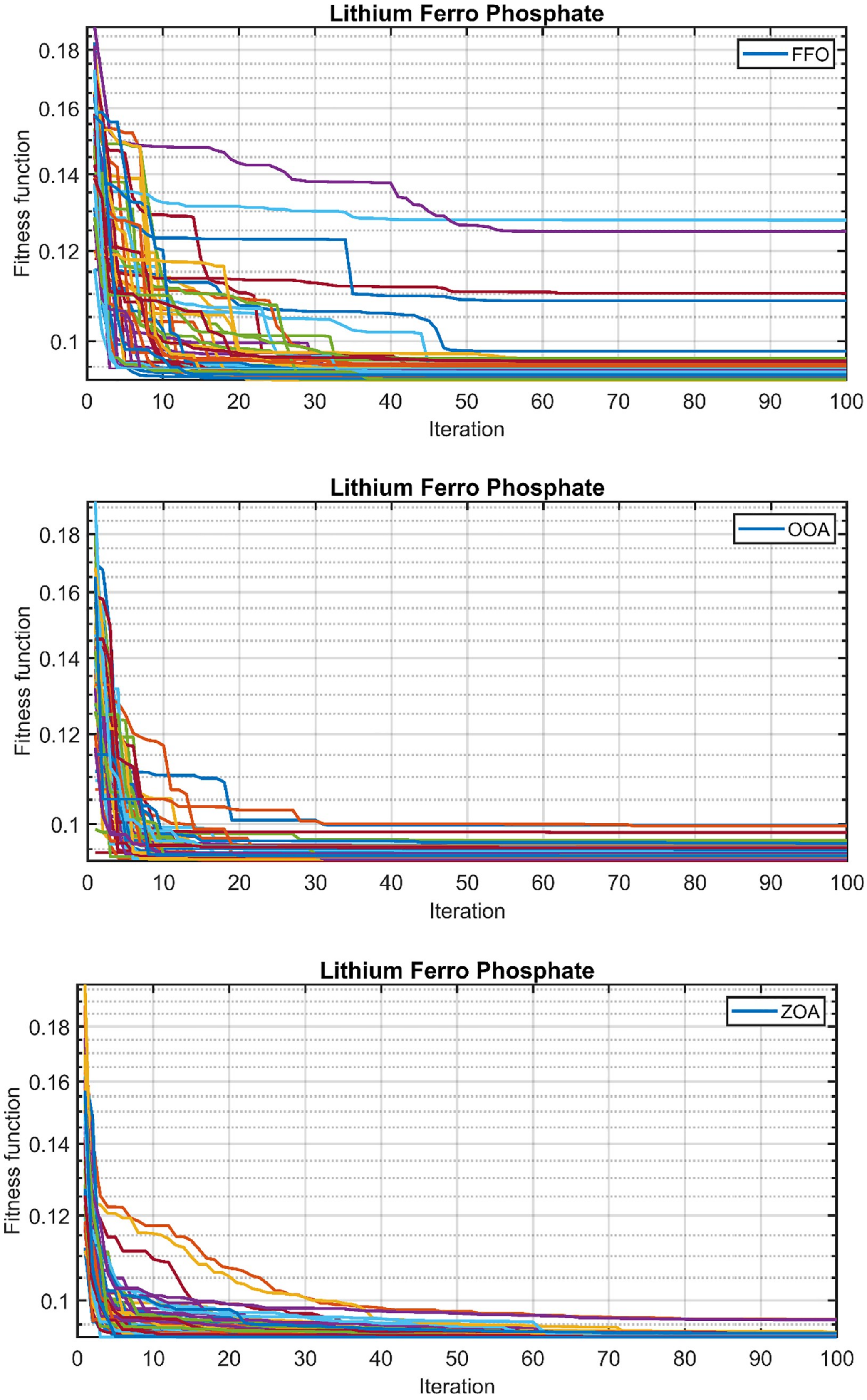

Fig 16. Fitness function over the 50 runs. (A) FFO. (B) OOA. (C) ZOA.

https://doi.org/10.1371/journal.pone.0317757.g016

Table 7. Results of the statistical analysis for case 2.

Statistical Metric	FFO	ZOA	OOA
**best**	0.09248081	0.09242801	0.09278760
**worst**	0.12765669	0.09604503	0.09976939
**mean**	0.09633949	0.09279498	0.09451994
**median**	0.09452575	0.09258548	0.09398435
**SD**	0.69291792	0.07063256	0.15187582
**RE**	2.08620932	0.19852045	0.93349730
**RMSE**	0.00787037	0.00078968	0.00229379

https://doi.org/10.1371/journal.pone.0317757.t007

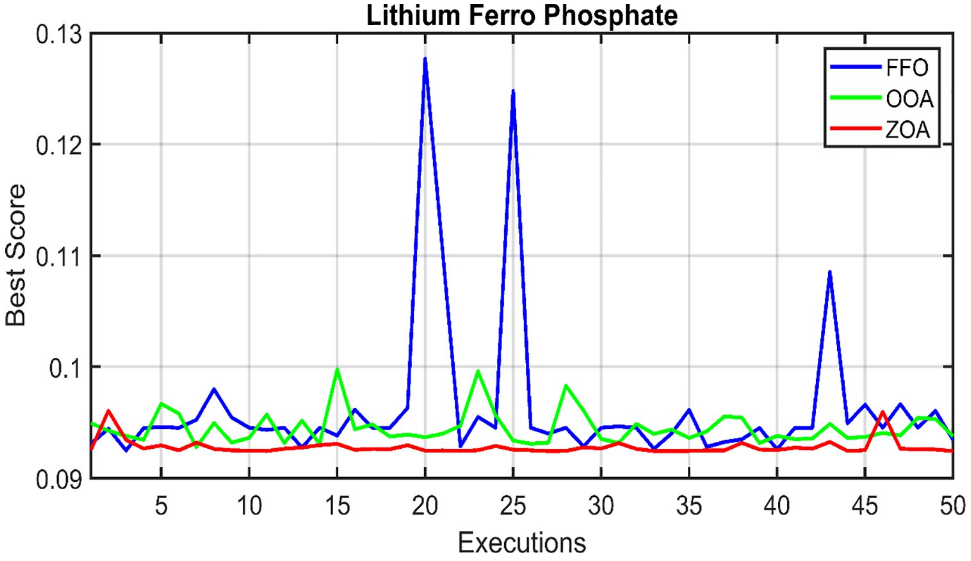

Fig 17. Final values of the convergence curves over the 50 runs.

https://doi.org/10.1371/journal.pone.0317757.g017

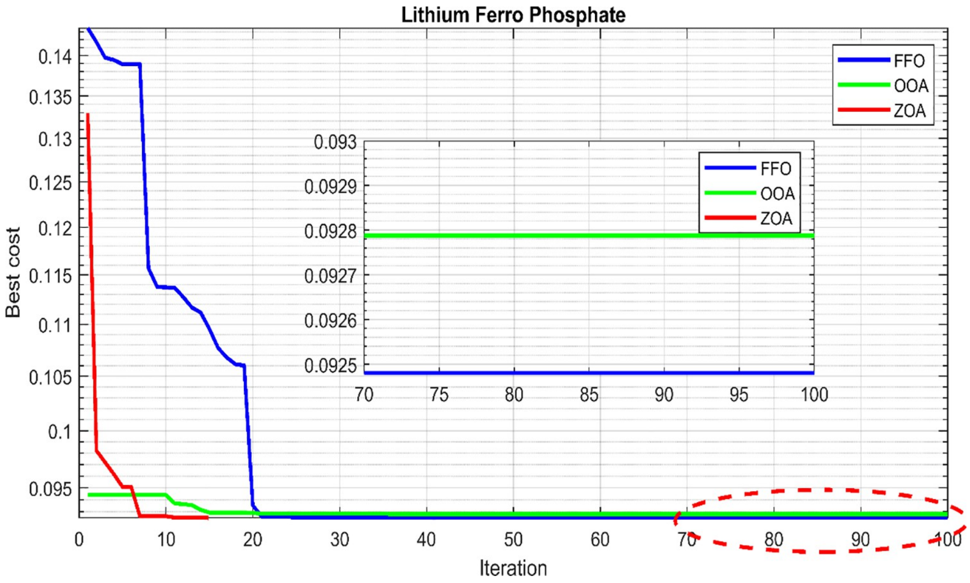

Fig 18. Convergence curves for case 2.

https://doi.org/10.1371/journal.pone.0317757.g018

Table 8. Optimization results of ZOA, OOA, and FFO for case 2.

Used Algorithm		FFO	ZOA	OOA
Best fitness		0.09248081	0.09242801	0.09278760
	N_{PV}	23.61300537	21.16611728	25.22585789
	N_{WT}	1.190177741	1.00037735	4.504424508
	N_{BG}	1.707274988	1.734804547	1.683222488
	N_{batt}	300	300.0001557	300.0381326
COE		0.204532969	0.202724864	0.206554621
NPC		5990866.441	5937906.185	6050081.572
LPSP		0.027634182	0.027778719	0.027874433

https://doi.org/10.1371/journal.pone.0317757.t008

Table 9. Annual cost distributed between system components for case 2.

	PV	WT	BG	Battery	Inverter
FFO	47209.23	765.0631264	54014.813	300335.2039	66321.50935
OOA	50433.79	2895.507937	53253.83936	300373.3791	66321.50935
ZOA	42317.19	643.0567442	54885.79396	300335.3597	66321.50935

https://doi.org/10.1371/journal.pone.0317757.t009

5. Conclusion and future work

This paper proposes an efficient methodology for designing an optimal hybrid system tailored for rural and isolated areas, leveraging the renewable resources prevalent in the region. The suggested approach focuses on optimizing the sizing of various technologies within the system to ensure a dependable energy supply while concurrently minimizing the cost of energy provision. In this study, three distinct battery technologies were evaluated: Flooded lead-acid, Lithium Ferro Phosphate, and Nickel Iron Battery. Our findings advocate for the consideration of Nickel Iron batteries in off-grid applications. To validate the effectiveness of our design approach, a case study was conducted in an actual village situated in Saudi Arabia, known for

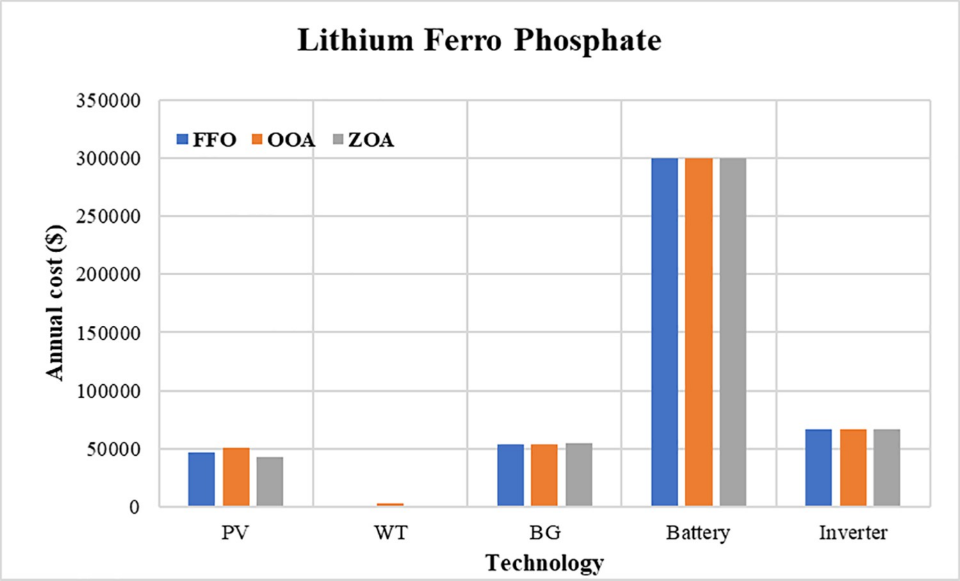

Fig 19. Annual cost divided between different subsystems for case 2.

https://doi.org/10.1371/journal.pone.0317757.g019

Table 10. Different types of cost distributed between system components for case 2.

	Used Algorithm	Capital Cost ($)	O&M Cost ($)	Replacement Cost ($)	Fuel Cost ($)
**PV**	**FFO**	30579.77	236.1300537	16393.32898	0
	**OOA**	32668.48	252.2585789	17513.05184	0
	**ZOA**	27410.96	211.6611728	14694.57692	0
**WT**	**FFO**	392.1408	119.0177741	253.9045848	0
	**OOA**	1484.122	450.4424508	960.943895	0
	**ZOA**	329.6052	100.037735	213.4138348	0
**BG**	**FFO**	2136.872	17500.25154	0	34377.68916
	**OOA**	2106.768	17253.7038	0	33893.36803
	ZOA	2171.329	17782.44053	0	34932.02436
Battery	FFO	181935.2	600	0	0
	OOA	181958.3	600.0762652	0	0
	ZOA	181935.3	600.0003113	0	0
Inverter	FFO	39645.51	0	26676	0
	OOA	39645.51	0	26676	0
	ZOA	39645.51	0	26676	0

https://doi.org/10.1371/journal.pone.0317757.t010

Table 11. Results of the statistical analysis for case 3.

Statistical Metric	FFO	ZOA	OOA
**best**	0.071212124	0.071212114	0.071623653
**worst**	0.091434080	0.071213845	0.090682102
**mean**	0.072891968	0.071212752	0.076871685
**median**	0.072676982	0.071212634	0.075598541
**SD**	0.315845569	0.000043838	0.451661903
**RE**	1.179465474	0.000447682	3.663616844
**RMSE**	0.003549395	0.000000771	0.006894468

https://doi.org/10.1371/journal.pone.0317757.t011

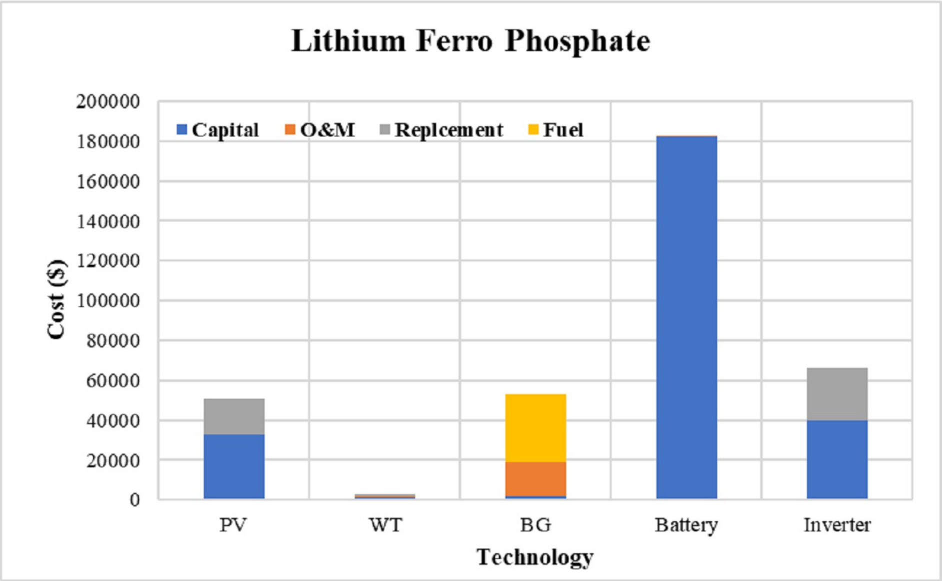

Fig 20. Division of annual cost with respect to cost type for case 2.

https://doi.org/10.1371/journal.pone.0317757.g020

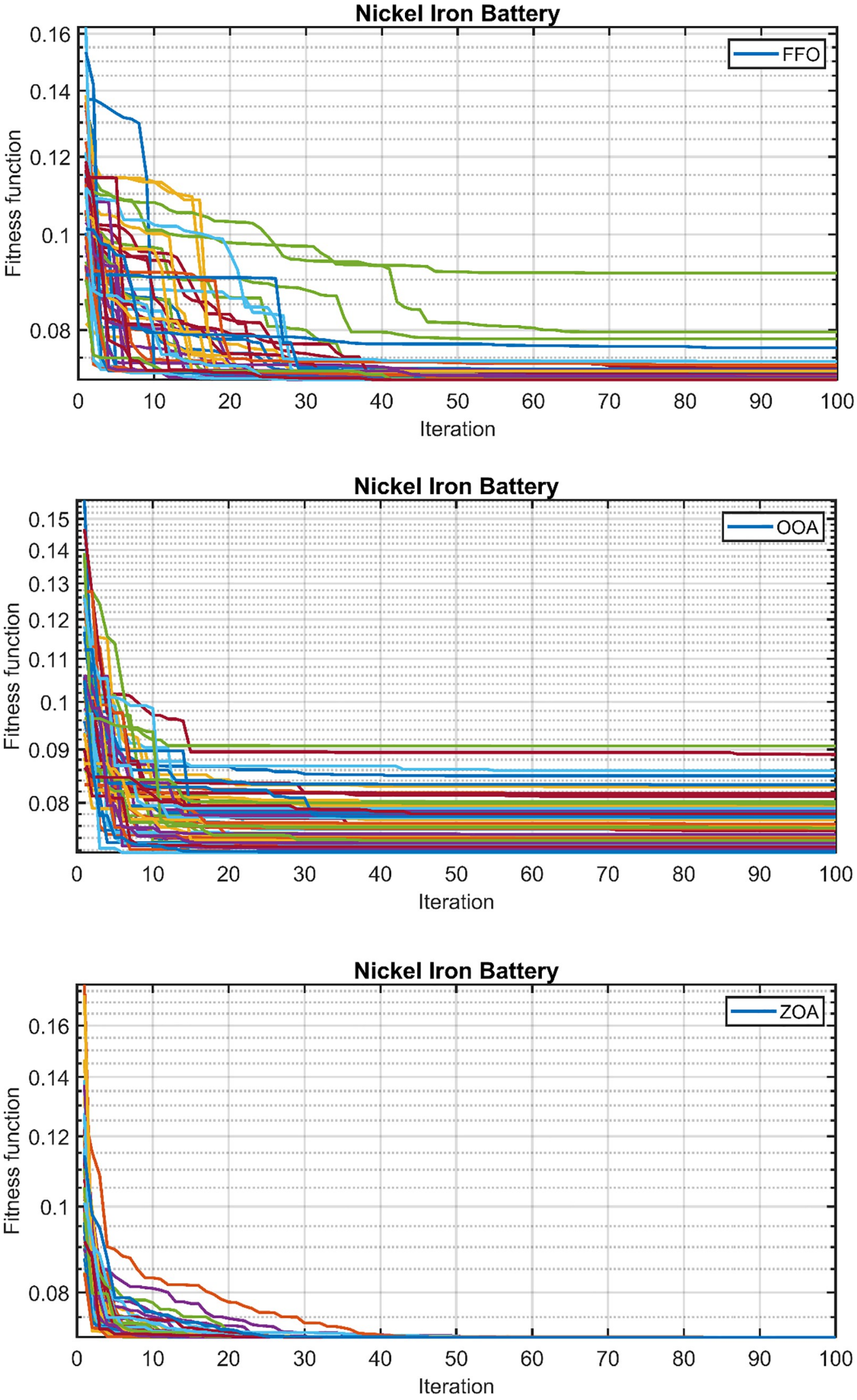

Fig 21. Fitness function over the 50 runs. (A) FFO. (B) OOA. (C) ZOA.

https://doi.org/10.1371/journal.pone.0317757.g021

Table 12. Optimization results of ZOA, OOA, and FFO for case 3.

Used Algorithm		FFO	ZOA	OOA
Best fitness		0.071212124	0.071212114	0.071623653
	N_{PV}	50	50.00000408	50.0000075
	N_{WT}	50	50.00032402	50.2551634
	N_{BG}	1.417251276	1.417240412	1.41535078
	N_{batt}	384.5977577	384.2830632	341.21979
COE		0.157954932	0.157912504	0.15215951
NPC		4626573.936	4625331.18	4456823.16
LPSP		0.018958869	0.018973922	0.02156155

https://doi.org/10.1371/journal.pone.0317757.t012

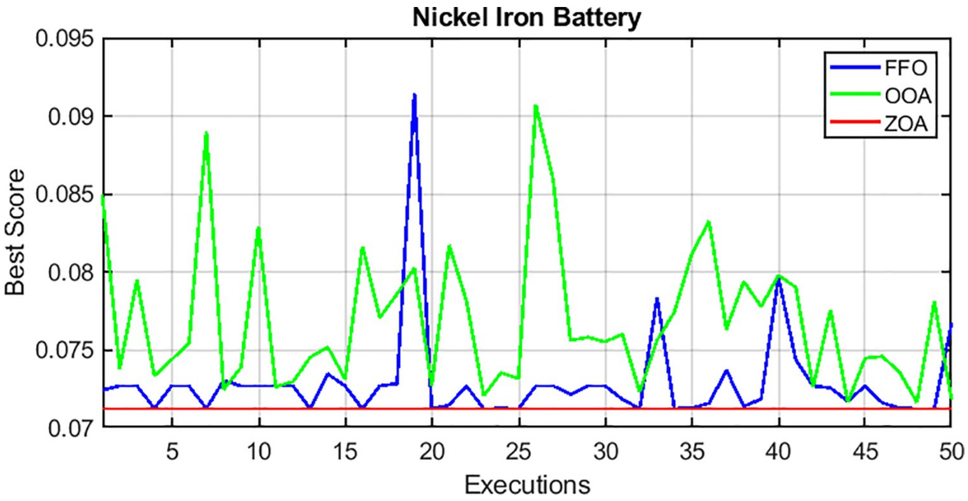

Fig 22. Final values of the convergence curves over the 50 runs.

https://doi.org/10.1371/journal.pone.0317757.g022

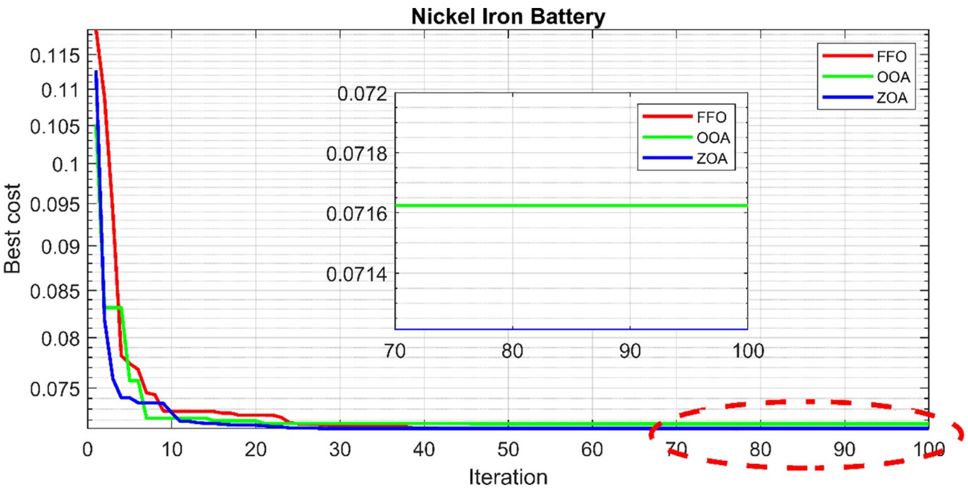

Fig 23. Convergence curves for case 3.

https://doi.org/10.1371/journal.pone.0317757.g023

Table 13. Annual cost distributed between system components for case 3.

Used Algorithm	PV	WT	BG	Battery	Inverter
FFO	99964.47	32140.7089	44839.03484	118655.972	66321.5093
OOA	99964.49	32304.7315	44778.9068	105273.0159	66321.5093
ZOA	99964.48	32140.9172	44838.69111	118558.8826	66321.5093

https://doi.org/10.1371/journal.pone.0317757.t013

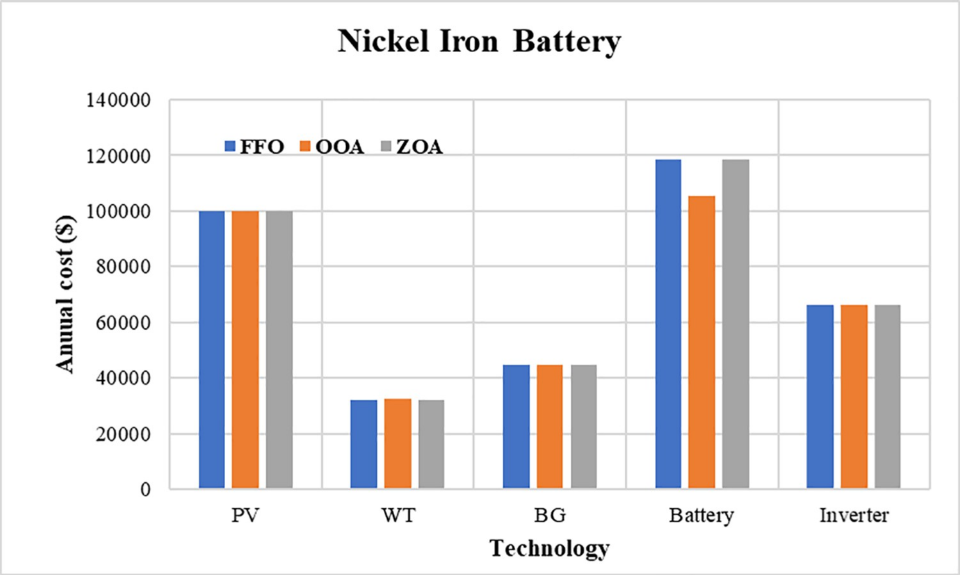

Fig 24. Annual cost divided between different subsystems for case 3.

https://doi.org/10.1371/journal.pone.0317757.g024

Table 14. Different types of cost distributed between system components for case 3.

	Used Algorithm	Capital Cost ($)	O&M Cost ($)	Replacement Cost ($)	Fuel Cost ($)
PV	FFO	64751.97	500	34712.5	0
	OOA	64751.98	500.000075	34712.50523	0
	ZOA	64751.98	500.000041	34712.50283	0
WT	FFO	16474.04	5000	10666.66667	0
	OOA	16558.11	5025.51634	10721.10152	0
	ZOA	16474.15	5000.0324	10666.73579	0
BG	FFO	1773.871	14527.3925	0	28537.7717
	OOA	1771.492	14507.9116	0	28499.50325
	ZOA	1773.857	14527.2811	0	28537.55293
Battery	FFO	116733	1922.98879	0	0
	OOA	103566.9	1706.09895	0	0
	ZOA	116637.5	1921.41532	0	0
Inverter	FFO	39645.51	0	26676	0
	OOA	39645.51	0	26676	0
	ZOA	39645.51	0	26676	0

https://doi.org/10.1371/journal.pone.0317757.t014

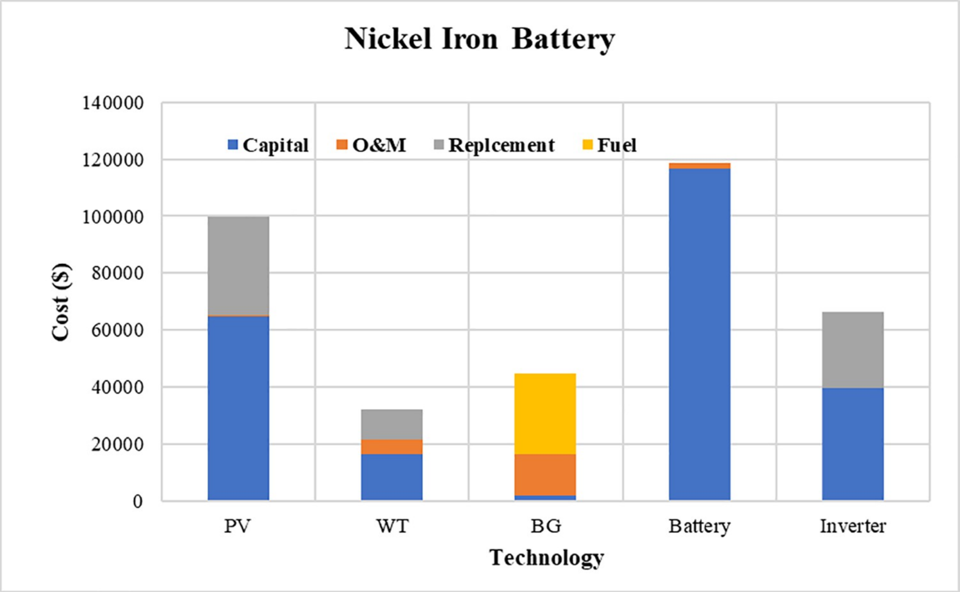

Fig 25. Division of annual cost with respect to cost type for case 3.

https://doi.org/10.1371/journal.pone.0317757.g025

its abundant sunlight and biomass resources. Accordingly, a PV/WT/Biomass/battery hybrid system was selected for this investigation. However, it's important to note that the proposed design framework is adaptable to other geographical locations, with adjustments made to accommodate the specific technological requirements dictated by the available resources. This study implements three optimization techniques: the Zebra Optimization Algorithm (ZOA), the Osprey Optimization Algorithm (OOA), and the Fly Foxes Optimization (FFO). The primary objective was to identify the most effective method for achieving the optimal sizing of the proposed hybrid system. Results indicate that the ZOA achieved the lowest electricity cost of 0.1285 $/kWh and a NPC of 3.8 M$, outperforming other optimization algorithms. Among

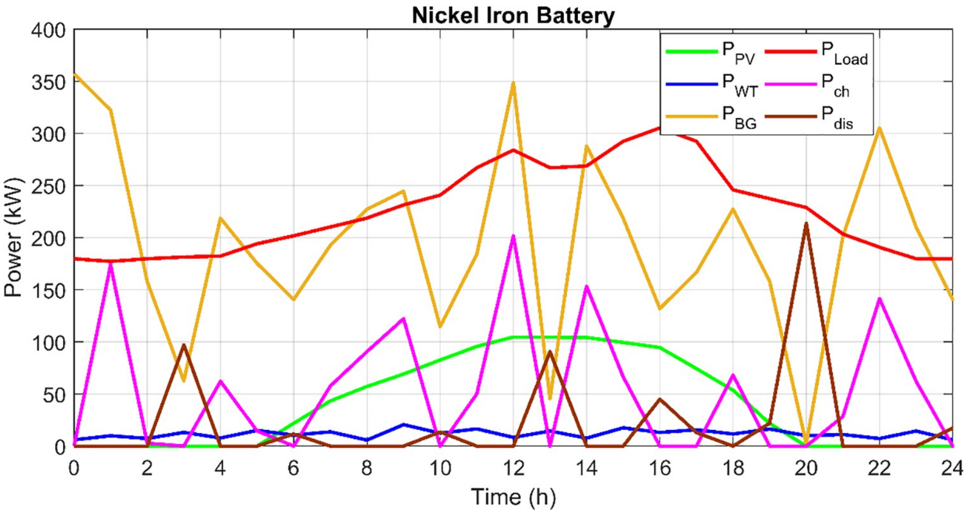

Fig 26. Variation of the system output according to the optimized parameters for case 3.

https://doi.org/10.1371/journal.pone.0317757.g026

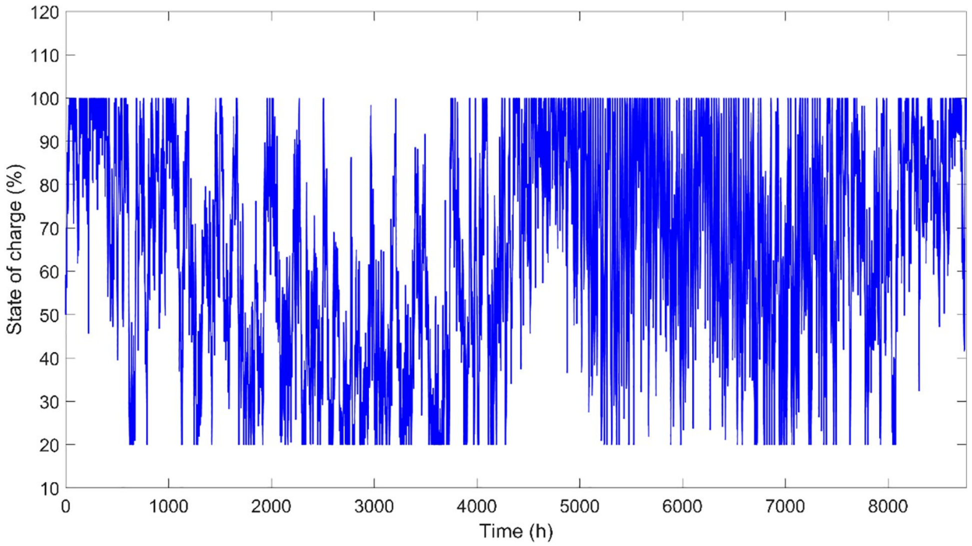

Fig 27. SOC of the battery system for case 3.

https://doi.org/10.1371/journal.pone.0317757.g027

the batteries tested, Nickel Iron (Ni-Fe) batteries provided the best cost-performance balance. These results highlight the economic viability and effectiveness of hybrid systems for off-grid applications.

Future work could focus on enhancing algorithmic performance by integrating machine learning techniques to improve convergence rates and accuracy in large-scale systems, as well as evaluating newer battery technologies, such as solid-state and lithium-sulfur batteries, for cost and efficiency gains. Additionally, advanced energy management strategies could be developed for dynamic load adjustments, improving the system's real-time responsiveness. Furthermore, sensitivity analysis on resource variability across regions would also help assess the robustness of the proposed model. Full life-cycle assessments of each component, particularly batteries, would quantify environmental impacts, and testing the model's scalability for larger or industrial loads would reveal its feasibility for diverse applications in off-grid settings.

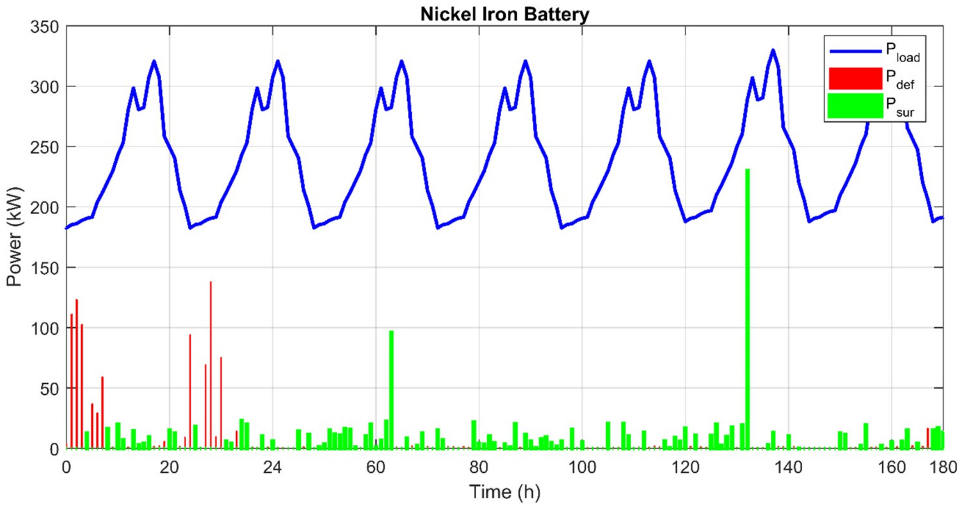

Fig 28. Hourly variation of load demand, surplus, and deficit power over a week for case3.

https://doi.org/10.1371/journal.pone.0317757.g028

Supporting information

S1 File.
(DOCX)

Author Contributions

Conceptualization: Mohammed Alqahtani.

Data curation: Mohammed Alqahtani.

Supervision: Muhammad Khalid.

Writing – original draft: Mohammed Alqahtani, Saeed Alhajri, Ahmed S. Menesy, Ali Maher Mohammed, Hamdy M. Sultan, Muhammad Khalid.

Writing – review & editing: Muhammad Khalid.

References

1. Sufyan M., Abd Rahim N., Tan C., Muhammad M. A., and Sheikh Raihan S. R., "Optimal sizing and energy scheduling of isolated microgrid considering the battery lifetime degradation," *PloS one*, vol. 14, no. 2, p. e0211642, 2019. https://doi.org/10.1371/journal.pone.0211642 PMID: 30763331
2. Rehman S. et al., "Design, commissioning and operation of a large-scale solar linear Fresnel system integrated with evacuated compound receiver: Field testing, thermodynamic analysis, and enhanced machine learning-based optimization," *Solar Energy*, vol. 278, p. 112785, 2024/08/01/ 2024.
3. Mohamed M. A., Eltamaly A. M., and Alolah A. I., "PSO-based smart grid application for sizing and optimization of hybrid renewable energy systems," *PloS one*, vol. 11, no. 8, p. e0159702, 2016. https://doi.org/10.1371/journal.pone.0159702 PMID: 27513000
4. Mussadiq U. et al., "The intelligent modelling and optimization of an economic and ecosystem-friendly model for grid connected prosumer community," *Plos one*, vol. 18, no. 1, p. e0276510, 2023. https://doi.org/10.1371/journal.pone.0276510 PMID: 36662811
5. Rehman S. et al., "Techno-economic evaluation and improved sizing optimization of green hydrogen production and storage under higher wind penetration in Aqaba Gulf," *Journal of Energy Storage*, vol. 99, p. 113368, 2024/10/10/ 2024.
6. Menesy A. S. et al., "A modified slime mold algorithm for parameter identification of hydrogen-powered proton exchange membrane fuel cells," *International Journal of Hydrogen Energy*, vol. 86, pp. 853–874, 2024/10/11/ 2024.
7. Brand B. and Fink T., "Renewable energy expansion in the MENA region: a review of concepts and indicators for a transition towards sustainable energy supply," 2014.
8. Cai W. et al., "Optimal sizing and location based on economic parameters for an off-grid application of a hybrid system with photovoltaic, battery and diesel technology," *Energy*, vol. 201, p. 117480, 2020.
9. Zhang W., Maleki A., Rosen M. A., and Liu J., "Sizing a stand-alone solar-wind-hydrogen energy system using weather forecasting and a hybrid search optimization algorithm," *Energy conversion and management*, vol. 180, pp. 609–621, 2019.
10. Lian J., Zhang Y., Ma C., Yang Y., and Chaima E., "A review on recent sizing methodologies of hybrid renewable energy systems," *Energy Conversion and Management*, vol. 199, p. 112027, 2019.
11. Akikur R. K., Saidur R., Ping H. W., and Ullah K. R., "Comparative study of stand-alone and hybrid solar energy systems suitable for off-grid rural electrification: A review," *Renewable and sustainable energy reviews*, vol. 27, pp. 738–752, 2013.
12. Pandiyan P. et al., "A comprehensive review of the prospects for rural electrification using stand-alone and hybrid energy technologies," *Sustainable Energy Technologies and Assessments*, vol. 52, p. 102155, 2022.
13. Bo G. et al., "Optimum structure of a combined wind/photovoltaic/fuel cell-based on amended Dragon Fly optimization algorithm: a case study," *Energy Sources*, *Part A*: *Recovery*, *Utilization*, *and Environmental Effects*, vol. 44, no. 3, pp. 7109–7131, 2022.
14. Al Essa M. J. M., "Energy assessments of a photovoltaic-wind-battery system for residential appliances in Iraq," *Journal of Energy Storage*, vol. 59, p. 106514, 2023.

15. Bakeer A., Elmorshedy M. F., Salama H. S., Elkadeem M. R., Almakhles D. J., and Kotb K. M., "Optimal design and performance analysis of coastal microgrid using different optimization algorithms," *Electrical Engineering*, vol. 105, no. 6, pp. 4499–4523, 2023.
16. Desai A., Mukhopadhyay I., and Ray A., "Solar PV-hydropower enhanced Picogrid as sustainable energy model for hilly remote areas: analytics and prospects thereof," *Energy Informatics*, vol. 6, no. 1, p. 24, 2023.
17. Ikram A. I., Shafiullah M., Islam M. R., and Rocky M. K., "Techno-Economic Assessment and Environmental Impact Analysis of Hybrid Storage System Integrated Microgrid," *Arabian Journal for Science and Engineering*, pp. 1–18, 2024.
18. Zhao X. and Zhang Y., "Integrated management of urban resources toward Net-Zero smart cities considering renewable energies uncertainty and modeling in Digital Twin," *Sustainable Energy Technologies and Assessments*, vol. 64, p. 103656, 2024.
19. E. Hamza, A. Iliasse, M. Ouardouz, and B. Abdes Samed, "Enhancing Local Energy Sharing Reliability within Peer-to-Peer Prosumer Communities: A Cellular Automata and Deep Learning Approach," *Abdennour and Ouardouz*, *Mustapha and Abdes Samed*, *Bernoussi*, *Enhancing* Local Energy Sharing Reliability within Peer-to-Peer Prosumer Communities: A Cellular Automata and Deep Learning Approach.
20. Zhong X., Sun X., and Wu Y., "A Bi-layer Optimization Method of the Grid-Connected Microgrid based on the Multi-strategy of Beluga Whale Algorithm," *Frontiers in Energy Research*, vol. 12, p. 1336205.
21. Tukkee A. S., bin Abdul Wahab N. I., binti Mailah N. F., and Bin Hassan M. K., "Optimal performance of stand-alone hybrid microgrid systems based on integrated techno-economic-environmental energy management strategy using the grey wolf optimizer," *Plos one*, vol. 19, no. 2, p. e0298094, 2024. https://doi.org/10.1371/journal.pone.0298094 PMID: 38330067
22. Ayed Y., Al Afif R., Fortes P., and Pfeifer C., "Optimal design and techno-economic analysis of hybrid renewable energy systems: A case study of Thala city, Tunisia," Energy Sources, Part B: Economics, Planning, and Policy, vol. 19, no. 1, p. 2308843, 2024.
23. Liu J., Li Y., Meng X., and Wu J., "Multi-Objective Optimization Based on Life Cycle Assessment for Hybrid Solar and Biomass Combined Cooling, Heating and Power System," *Journal of Thermal Science*, pp. 1–20, 2024.
24. Zaman M. R., "An Intensive Analysis of the Energy Management System for Hybrid Electric Vehicles and Electric Drive System Powered By Renewable Energy Sources," *Control Systems and Optimization Letters*, vol. 2, no. 1, 2024.
25. Samy M. M. and Barakat S., "Hybrid invasive weed optimization-particle swarm optimization algorithm for biomass/PV micro-grid power system," in 2019 21st international Middle East power systems conference (MEPCON), 2019, pp. 377–382: IEEE.
26. Barakat S., Samy M., Eteiba M. B., and Wahba W. I., "Viability study of grid connected PV/Wind/Biomass hybrid energy system for a small village in Egypt," in *2016 eighteenth international Middle East power systems conference (MEPCON)*, 2016, pp. 46–51: IEEE.
27. Samy M., Sarhan H., Barakat S., and Al-Ghamdi S., "A hybrid pv-biomass generation based micro-grid for the irrigation system of a major land reclamation project in kingdom of saudi arabia (ksa)-case study of albaha area," in *2018 IEEE International Conference on Environment and Electrical Engineering and 2018 IEEE Industrial and Commercial Power Systems Europe (EEEIC/I&CPS Europe)*, 2018, pp. 1–8: IEEE.
28. Alshammari N., Samy M. M., and Asumadu J., "Optimal economic analysis study for renewable energy systems to electrify remote region in Kingdom of Saudi Arabia," in *2018 Twentieth International Middle East Power Systems Conference (MEPCON)*, 2018, pp. 1040–1045: IEEE.
29. J. M. Idris, Y. K. Abubakar, A. Nasir, A. M. Zumami, B. Bongfa, and D. J. Adelodun, "Techno-Economic and Emission Analysis of Biomass Integrated Hybrid Energy System (Hes) for an Automotive Condition Monitoring Centre," Available at SSRN 4726908.
30. Modu B., Abdullah M. P., Alkassem A., Al Garni H. Z., and Alkabi M., "Optimal Design of a Grid-Independent Solar-Fuel Cell-Biomass Energy System Using an Enhanced Salp Swarm Algorithm Considering Rule-Based Energy Management Strategy," *IEEE Access*, 2024.
31. Menesy A. S. et al., "Techno-economic optimization framework of renewable hybrid photovoltaic/wind turbine/fuel cell energy system using artificial rabbits algorithm," *IET Renewable Power Generation*, 2024.
32. Sultan H. MMenesy A. S., Kamel S., Alghamdi A. S., and Zohdy M., "Optimal sizing of isolated hybrid PV/WT/FC system using manta ray foraging optimization algorithm," *Int. Trans. J. Eng. Manag. Appl. Sci. Technol*, vol. 11, pp. 1–12, 2020.

33. Sultan H. M., Menesy A. S., Kamel S., Korashy A., Almohaimeed S., and Abdel-Akher M., "An improved artificial ecosystem optimization algorithm for optimal configuration of a hybrid PV/WT/FC energy system," *Alexandria Engineering Journal*, vol. 60, no. 1, pp. 1001–1025, 2021.

34. H. M. Sultan, A. S. Menesy, S. Kamel, A. S. Alghamdi, and C. Rahmann, "Optimal design of a grid-connected hybrid photovoltaic/wind/fuel cell system," in 2020 IEEE Electric Power and Energy Conference (EPEC), 2020, pp. 1–6: IEEE.

35. Menesy A. S., Sultan H. M., Habiballah I. O., Masrur H., Khan K. R., and Khalid M., "Optimal Configuration of a Hybrid Photovoltaic/Wind Turbine/Biomass/Hydro-Pumped Storage-Based Energy System Using a Heap-Based Optimization Algorithm," *Energies*, vol. 16, no. 9, p. 3648, 2023.

36. Toopshekan A., Rahdan P., Rad M. A. V., Yousefi H., and Astaraei F. R., "Evaluation of a stand-alone CHP-Hybrid system using a multi-criteria decision making due to the sustainable development goals," *Sustainable Cities and Society*, vol. 87, p. 104170, 2022.

37. Kharrich M. et al., "Developed approach based on equilibrium optimizer for optimal design of hybrid PV/wind/diesel/battery microgrid in Dakhla, Morocco," *IEEE Access*, vol. 9, pp. 13655–13670, 2021.

38. Trojovská E., Dehghani M., and Trojovský P., "Zebra optimization algorithm: A new bio-inspired optimization algorithm for solving optimization algorithm," *IEEE Access*, vol. 10, pp. 49445–49473, 2022.

39. Dehghani M. and Trojovský P., "Osprey optimization algorithm: A new bio-inspired metaheuristic algorithm for solving engineering optimization problems," *Frontiers in Mechanical Engineering*, vol. 8, p. 1126450, 2023.

40. Zervoudakis K. and Tsafarakis S., "A global optimizer inspired from the survival strategies of flying foxes," *Engineering with Computers*, pp. 1–34, 2023.
